# Targeting CLEC4E in immunosuppressive tumour‐associated macrophages via BET inhibition

**DOI:** 10.1002/ctm2.70505

**Published:** 2025-10-15

**Authors:** Mengting Liao, Kexin Long, Liang Dong, Zhuo Li, Wenhua Wang, Rui Hu, Yangyi Zhang, Juan Su, Wu Zhu, Xiang Chen, Mingzhu Yin

**Affiliations:** ^1^ Department of Dermatology Hunan Key Laboratory of Skin Cancer and Psoriasis Hunan Engineering Research Center of Skin Health and Disease Xiangya Hospital Central South University Changsha China; ^2^ National Engineering Research Center of Personalized Diagnostic and Therapeutic Technology Changsha China; ^3^ Health Management Center Xiangya Hospital Central South University Changsha China; ^4^ National Clinical Research Center for Geriatric Diseases (Xiangya Hospital) Changsha China; ^5^ Clinical Research Center Medical Pathology Center Cancer Early Detection and Treatment Center and Translational Medicine Research Center Chongqing University Three Gorges Hospital Chongqing University Chongqing China

**Keywords:** BET inhibitor, CLEC4E, conditional knockout mice, single‐cell RNA sequencing, tumour‐associated macrophage

## Abstract

**Background:**

Immunosuppressive tumour‐associated macrophages (TAMs) represent a promising target for cancer immunotherapy; however, existing TAM‐directed therapies have shown limited clinical efficacy. C‐type lectin domain family 4 member E (CLEC4E), a pro‐inflammatory molecule expressed on macrophages, was recently found to be highly enriched in TAMs. This study aims to elucidate the role of CLEC4E in TAMs and identify potential therapeutic agents targeting CLEC4E, and to clarify the mechanism of Bromodomain and extraterminal domain (BET) inhibitor NHWD‐870 in downregulating CLEC4E.

**Methods:**

We first assessed the correlation between CLEC4E expression and survival in melanoma patients. Clec4e^flox/flox^ Lyz2‐cre (knockout) and Clec4e^flox/flox^ (control) mice were generated and implanted with melanoma or ovarian cancer models. Single‐cell RNA sequencing was performed to characterise macrophage phenotypic changes following CLEC4E knockout, with validation via RT‐PCR, flow cytometry and proteomic sequencing. A drug screen identified BET inhibitors targeting CLEC4E, and their mechanisms were further investigated using RNA silencing, Chromatin Immunoprecipitation (ChIP)‐seq and luciferase reporter assays.

**Results:**

In melanoma patients, high CLEC4E^+^ TAM infiltration was associated with poor prognosis. CLEC4E knockout significantly suppressed tumour growth compared to control mice. TAMs from knockout mice exhibited downregulated proliferation markers and upregulated genes related to antigen presentation and pro‐inflammatory responses. Mechanistically, CLEC4E deletion inhibited TAM proliferation via the Erk signalling pathway, enhanced TAM‒T cell interactions, and increased granzyme B expression in T cells. The BET inhibitor NHWD‐870 was shown to disrupt BRD4‒CEBPβ interaction, leading to downregulation of CLEC4E expression.

**Conclusions:**

CLEC4E^+^ TAMs promote an immunosuppressive microenvironment by enhancing their own proliferation and impairing anti‐tumour functions, thereby limiting T‐cell cytotoxicity. Targeting the BRD4/CEBPβ/CLEC4E axis with BET inhibitors represents a promising therapeutic strategy for reprogramming TAMs and enhancing anti‐tumour immunity.

## Graphical abstract

NHWD‐870 as a BET inhibitor holds the potential to serve as a tumour‐associated macrophages targeted therapy through promoting macrophages proliferation and TAM‒T cell interactions by suppressing BRD4/CEBPβ/CLEC4E axis.

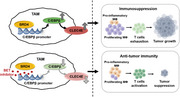

## 1 INTRODUCTION

Tumour‐associated macrophages (TAMs) are a key component of the tumour microenvironment (TME), where they typically exert immunosuppressive functions and promote tumour progression.^1^, ^2^ Their abundance is frequently associated with poor prognosis across multiple cancer types.^3^, ^4^, ^5^ In recent years, therapeutic strategies targeting TAMs have emerged, including approaches of depleting TAM populations, inhibiting their recruitment, blocking their pro‐tumour activities, or restoring the anti‐tumour effect of TAMs.^1^, ^6^, ^7^ Several TAM‐targeting agents have shown promising results in preclinical studies and are currently under clinical evaluation.^8^ However, while some of these drugs exhibit encouraging anti‐tumour activity in early‐phase clinical trials, others fail to elicit objective clinical responses.^9^, ^10^, ^11^, ^12^ Therefore, there remains a need to uncover novel targets involved in regulating the immunosuppressive behaviour of TAMs.

C‐type lectin domain family 4 member E (CLEC4E), which encodes the macrophage‐inducible C‐type lectin (Mincle), is a transmembrane molecule predominantly expressed on macrophages.^13^ Upon ligand binding, CLEC4E triggers the secretion of cytokines and other immune mediators in the inflammation.^14^ It has been well established that CLEC4E plays a pro‐inflammatory role in infectious diseases, autoimmune disorders as well as tissue injury.^15^, ^16^, ^17^, ^18^, ^19^

Nonetheless, the role of CLEC4E under the context of tumour remains poorly understood. Limited literature suggests that CLEC4E is related to the immune landscape of TME in melanoma and exhibit pro‐tumour effect by promoting an M2‐like phenotype in TAMs.^20^, ^21^ Elevated CLEC4E expression in TAMs has also been implicated in the progression of gastric cancer (GC).^22^ Interestingly, by RNA sequencing data our preclinical studies revealed significant upregulation of CLEC4E in TAMs. Although our previous work established a negative correlation between TAM infiltration and patient prognosis,^23^ the specific mechanisms by which CLEC4E mediates the TME and contributes to tumour progression have not been elucidated yet.

The BET protein family, comprising BRD2, BRD3, BRD4 and BRDT, are epigenetic ‘readers’, which bind to acetylated lysine and modulate gene transcription.^24^ BET inhibitors (BETi), including NHWD‐870 developed by our team, effectively suppress the proliferation and metastasis of tumour cells by inhibiting BRD4 expression. In preclinical studies, NHWD‐870 has been shown to induce tumour regression in models of ovarian cancer, osteosarcoma, diffuse large B‐cell lymphoma and melanoma.^23^, ^25^, ^26^, ^27^, ^28^, ^29^ Beyond their direct effects on tumour cells, BETi also promote favourable changes in immune cell populations within the TME, such as reducing TAMs and dendritic cells, and enhancing cytotoxic T‐cell infiltration.^30^, ^31^, ^32^ However, the mechanisms through which BETi modulate immune cell function remains to be explored.

In this study, we developed the first myeloid‐specific CLEC4E knockout mouse model and demonstrated the pro‐tumoural role of macrophage‐expressed CLEC4E in multiple tumour types. With single‐cell RNA sequencing (scRNA‐seq), we revealed a previously unknown mechanism through which CLEC4E^+^ TAMs exert immunosuppressive effects within the tumour microenvironment. Furthermore, we identified BETi as a potent suppressor of CLEC4E expression, thereby uncovering a new immune‐regulatory mechanism of BET inhibition and proposing a novel TAM‐targeted therapeutic strategy for cancer treatment.

## 2 MATERIAL AND METHODS

### 2.1 Patient sample

The study was approved by the Institutional Review Board of Xiangya Hospital, Central South University, and written informed consent was obtained from all enrolled patients. Tissues were removed from melanoma patients in the department of dermatology at Xiangya Hospital, who received tumour excision or biopsy between January 2017 and May 2021. Thirty‐three tumour tissues were obtained for survival analysis. Six paired tumour and tumour adjacent (within 2 cm from tumour) tissues were obtained.

### 2.2 Mouse model

Wild‐type C57BL/6 mice were purchased from Hunan SJA Laboratory Animal Co., Ltd. Clec4e^flox/+^ (C57BL/6J‐Clec4e^em1cyagen^) and Lyz2‐cre mice were purchased from Cyagen Biosciences. Clec4e^flox/flox^ Lyz2‐cre mice (Lyz2 ΔCLEC4E) were generated to selectively disrupt Clec4e gene in the cells of the myeloid lineage. Knockout and breeding strategies are shown in Figure S2a,b. Male mice at 8‒10 weeks were randomly divided into groups of five mice each, and the experimental group was subcutaneously injected with 5 × 10^5^ B16F10 cells for melanoma model, and mice were sacrificed when the largest tumour reached 1500 mm^3^. Female mice at 8‒10 weeks were randomly divided into groups of seven mice each, and the experimental group were intraperitoneal injected with 1 × 10^6^ ID8 cells for ovarian model, and mice were sacrificed 18 weeks after injection. During the experimental process, potential confounders (the order of treatments and measurements, and location of animals/cages) were controlled through randomisation.

### 2.3 scRNA‐seq analysis

Fresh tumour tissues were collected from Clec4e^flox/flox^ Lyz2‐cre and control mice, and washed with phosphate‐buffered saline (PBS). Five tumours from each group were collected, mixed and treated as one sample. Tissue was cut into 1 mm^3^ pieces and incubated in the dispase (Sigma, D4693) solution at 37°C for 45 min. Then, the tissue was gently dissociated with a pipette and incubated in trypsin .05% solution diluted with PBS for 5 min. After the trypsin was deactivated with 5% foetal bovine serum (FBS), the samples were filtered out with a 40 µm filter. Single cells were counted with automated cell counters (Countstar Rigel S3). Live cells were preferentially sorted for single‐cell sequencing.

Singleron platform was used in our study. Single‐cell suspensions (1 × 10^5^ cells/mL) with PBS were loaded into microfluidic devices using the Singleron Matrix Single Cell Processing System (Singleron). Subsequently, the scRNA‐seq libraries were constructed according to the protocol of the GEXSCOPE Single Cell RNA Library Kits (Singleron).^33^ Individual libraries were diluted to 4 nM and pooled for sequencing. At last, pools were sequenced on Illumina HiSeq X with 150 bp paired end reads.

Seurat (version 3.0.1) was used for the procession QC. Cells with <300 unique molecular identifiers (UMIs) in a single cell or >15% of mitochondrion‐derived UMI counts were considered low‐quality cells and removed. The top 20 principal components, along with the first 2000 variable genes, were used in this process. Then, the influence of the UMI count and the percentage of mitochondrion‐derived UMI counts were regressed out with the ScaleData function. Subsequently, the main cell clusters were identified with the FindClusters function of Seurat. To precisely annotate the cell types, we manually curated gene markers or each cell type.

The Monocle 2 package (v.2.8.0) was used to analyse single‐cell trajectories in order to discover the cell state transitions with the following parameters: num_cells_expressed 10, qval < .01 (differentialGeneTest function). The trajectory was visualised as a 2D tSNE graph.

Differentially expressed genes (DEGs) were identified by using the FindMarkers function embedded in Seurat with the following cutoff thresholds: Benjamini‒Hochberg adjusted *p*‐value <.01 and fold change (FC) >.5. Then, these DEGs were loaded into the clusterProfiler package for the GO enrichment analysis. Pathways with adjusted *p*‐values <.05 were considered significantly enriched.

The CellChat (V1.1.3, https://github.com/sqjin/CellChat) algorithm was used to infer cell‒cell interactions between TAMs and T cells and identify differential interactions between Clec4e^flox/flox^ Lyz2‐cre and control samples. We followed the official workflow and imported gene expression data into CellChat using ‘createCellChat’ function. We mainly applied ‘identifyOverExpressedGenes’, ‘identifyOverExpressedInteractions’ and ‘projectData’ functions to detect significant cell‒cell interactions among the investigated cells. Ligand‒receptor pairs with thresh.pc (threshold of the percent of cells expressed in one cluster) >.1 and thresh.fc (threshold of log FC) >.1. All cell interaction visualisations were plotted using the CellChat package.

### 2.4 Cell culture

B16F10, ID8, THP‐1, A375, SK‐MEL‐28, WM35, RAW264.7 and A2780 cells were purchased from American Type Culture Collection. B16F10 and THP‐1 were cultured in Dulbecco's modified Eagle medium (DMEM); ID8, A375, SK‐MEL‐28, WM35, RAW264.7 and A2780 were cultured in RPMI‐1640 medium. Medium was supplemented with 10% FBS (Gibco) and 1% antibiotics (penicillin/streptomycin). THP‐1 cells were seeded in the plate with 100 ng/mL (Phorbol myristate acetate) PMA (Sigma‒Aldrich, P8139) and cultured for 24 h to induce macrophages. PMA was replaced with fresh medium before further treatment.

Bone marrow‐derived macrophages (BMDMs) were obtained from C57BL/6 mice. Briefly, the skin and muscle of lower limbs were removed, and ends of femur and tibia were cut off. Bone marrow was flushed into Petri dish with Iscove's modified Dulbecco medium (IMDM) supplemented with 20 ng/mL M‐CSF (Peprotech), 10% FBS (Gibco), 1% non‐essential amino acids (Gibco), 1% sodium pyruvate (BI), 1% penicillin/streptomycin and 45 µM β‐mercaptoethanol. Cells were cultured for 5‒7 days when BMDM were further used.

For peritoneal macrophage, 8‒12 weeks C57BL/6 mice were intraperitoneal injected with 2 mL 3% thioglycolate medium (Sigma, T9032) for 3 days, and peritoneal lavage fluid was collected, which contained macrophages. Cells were cultured in DMEM with 10% FBS and 1% antibiotics for 2 days, and suspended cells were washed. Adherent cells were digested with trypsin and plated for further use.

### 2.5 In vitro induction of TAM

TAMs were induced by culturing macrophages with tumour cell conditioned medium. Briefly, B16F10, A375, SK‐MEL‐28, WM35 or A2780 cells were cultured for 24‒48 h until 100% confluency when supernatant was collected and filtered with .22 µm filter. Supernatant was added to macrophages along with drug treatment as indicated in the result. RAW264.7 cells were cultured for 24 h, and THP‐1 cells for 48 h to induce TAM.

### 2.6 siRNA transfection

Transfection of BMDM and THP‐1 was performed using siRNA (mouse BRD2, BRD3, BRD4, CLEC4E), purchased from RIBOBIO; siRNA (mouse CEBPB and human BRD4) from GenePharma; and Lipofectamine RNAiMAX (ThermoFisher). Cells were seeded in six‐well plate and transfected with 3 pmol siRNA and 7.5 µL Lipofectamine RNAiMAX per well, incubated for 24 h. Medium was replaced with tumour conditioned medium to induce TAM. After another 24 h, cells were harvested for RNA or protein extraction.

### 2.7 RT‐PCR

Total RNA was extracted from indicated cells using Magzol Reagent (Magen, R4801) according to manufacturer's instructions, and cDNA was synthesised using HiScript Q RT Reverse Transcription Kit (Vazyme, R223‐01). RT‐PCR was performed with ABI QuantStudio 3 using the 2× SYBR Green qPCR Master Mix (bimake, B21703). Relative expression levels were determined by normalising GAPDH or ACTB using ΔΔCt method. Primer sequences are listed in Table S1.

### 2.8 RNA sequencing

Peritoneal macrophages were isolated and cultured in IMDM (M0), or treated with B16 conditioned medium to induce TAM as described above. M0 and TAM were subjected to RNA sequencing to screen for gene enrichment in TAM. With TAM, Clec4e was silenced with siRNA and efficiency was verified with RT‐PCR as above, followed by another set of RNA sequencing. Sequencing was performed using MGIseq‐2000 in Shenzhen BGI Technology Service Co., Ltd.

### 2.9 Western blotting

Cells were homogenised in RIPA lysis buffer (Beyotime, P0013C). The lysates were incubated on ice for 30 min and centrifuged at 14 000 rpm for 10 min at 4°C. Supernatants were collected and the protein concentration was determined with a BCA Protein Assay Kit (CWBIO, CW0014S). The lysates were then subjected to SDS‒PAGE followed by immunoblotting with specific antibodies, detected using a Western Blot Detection Kit (Kindle Biosciences, R1004). All antibodies used for Western blotting are listed in Table S2.

### 2.10 Proteome sequencing

Peritoneal macrophages were isolated from Lyz2 ΔCLEC4E and control mice, and total protein was extracted as described above. Proteome and phosphoproteome sequencing were performed using Bruker tims‐TOF Pro in Hangzhou Jingjie Biotechnology Co., Ltd.

### 2.11 Immunostaining

Immunofluorescence staining and immunohistochemical of tumour sections were performed according to the manufacturer's instructions. Antibodies used for immunostaining are listed in Table S2. Slides were imaged using a Nikon Eclipse Ti2 Microscope and Nikon Intensilight C‐HGFI Illuminator, and images were captured using NIS‐Elements D 5.01.00 Software. Positive cell count and mean fluorescence intensity measurement were evaluated with ImageJ.

### 2.12 Flow cytometry and cell sorting

Flow cytometry analyses were performed using antibodies listed in Table S2. Cells were stained for fixable viability dye to identify live/dead cells. Surface marker (CD45, CD11b, F4/80, CD3, CD4, CD8) antibodies for 20 min at 4°C. For intracellular staining, cells were permeabilised with Transcription Factor Staining Buffer Set (eBioscience, 00‐5523‐00) according to manufacturer's instructions, and stained with CD68, CD206, Granzyme B or Ki67 for 30 min at 4°C. Flow cytometry was performed on a FACS LSRFortessa (BD Biosciences). Data were analysed with Flowjo software (version 10.0.7). For macrophage sorting, cells were stained with CD45, F4/80 and CD11b, and positive cells were collected using BD FACSMelody (BD Biosciences).

### 2.13 Cell counting kit‐8 assay

Cell proliferation was analysed by cell counting kit‐8 (CCK‐8, Selleck, B34302) according to the manufacturer's protocols. Cells were seeded at 5000 cells per well with IMDM in 96‐well plates. After 24 h, cells were washed and treated with fresh DMEM medium for M0 (RAW264.7), IMDM for M0 (BMDM) group, or B16 conditioned medium for TAM (BMDM) group and TAM (RAW264.7) group. Add ERK1/2 inhibitor (Selleck, S7101) to the cells differentiated from RAW264.7 according to experimental grouping. At the present time point, cell counting assay was performed for all groups of cells.

### 2.14 CHIP sequencing

A375 cells were treated with BET inhibitor NHWD‐870 (4 nM) or (Dimethyl sulfoxide) DMSO for 72 h, or transfected with siRNA (BRD4) or negative control, then harvested and stored at ‒80°C. CHIP sequencing with BRD4 antibody (Cell Signaling Technology, #13440) and data analysis was performed in Acegen Company. Data visualisation was performed with Integrative Genomics Viewer.

### 2.15 Dual‐luciferase reporter gene assay

pGL3‐Basic and pGL3‐hCLEC4E‐promoter plasmids with firefly luciferase were purchased from Sangon Biotech (Shanghai). pcDNA3.1(+) and pcDNA3.1(+)‐hBRD4 were purchased from GenePharma. pRL‐TK was purchased from Fenghui Biotechnologies. Briefly, 293T cells were cultured in 24‐well plate until 50% confluency, when pRL‐TK, pGL3 and pcDNA3.1(+) plasmids were co‐transfected with Lipofectamine 2000 transfection reagent (ThermoFisher, 11668019). After 24 h of culture, cells were lysed and subjected to luciferase activity evaluation with the Dual Luciferase Assay Kit (Promega, E1910) according to the manufacturer's instructions. DX_lumi_880 system was utilised to measure the *firefly* luciferase activity followed by normalising to the *Renilla* luciferase activity.

### 2.16 Statistical analysis

Statistical analyses were performed with GraphPad Prism (version 8.3.0), where data were analysed using two‐tailed Student's *t*‐tests. *p*‐Values less than .05 were considered statistically significant. Overall survival of patients was defined as the interval from the date of primary surgery or biopsy to the date of death. The log‐rank test was used to compare survival outcomes between different groups of patients.

Gene expression profiling interactive analysis (http://gepia2.cancer‐pku.cn/#index) was used to analyse the RNA sequencing data of tumours and normal tissues from TCGA database. Differential expressions of CLEC4E in skin carcinoma and normal skin tissue were plotted. The correlations of CLEC4E with CD14, CD68, S100B and MIA expression in tumour samples were analysed.

## 3 RESULTS

### 3.1 Enrichment of CLEC4E in TAMs is associated with unfavourable patient survival

**FIGURE 1:**
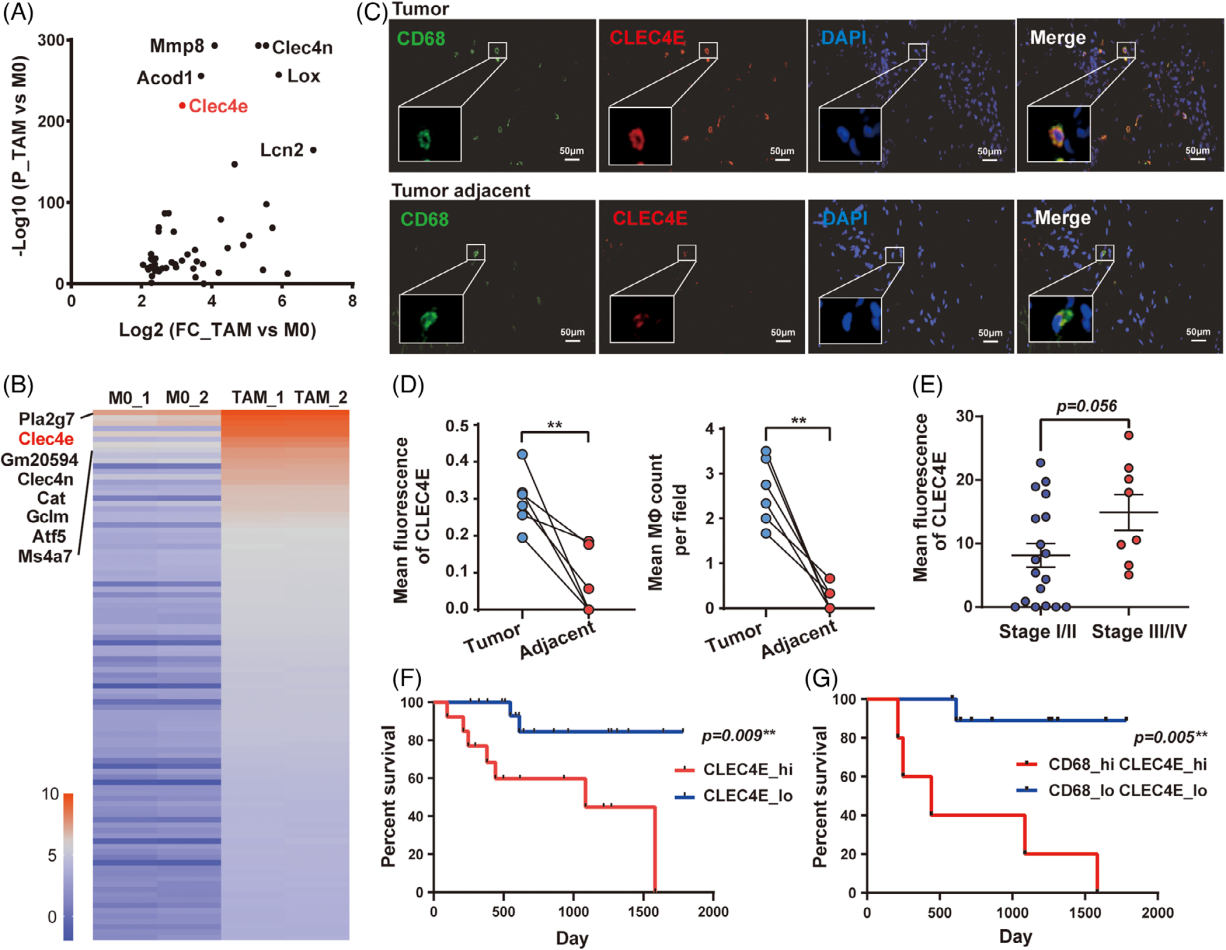
Enrichment of C‐type lectin domain family 4 member E (CLEC4E) in tumour‐associated macrophage (TAM) is correlated with unfavourable patient prognosis. (A) Volcano plot of gene enrichment in TAMs by RNA sequencing. (B) Heatmap of gene expressions in M0 and TAM by RNA sequencing, ranked by expression level in TAM. (C) Immunofluorescence of paired tumour and tumour adjacent tissues from melanoma patients. (D) Comparison of CLEC4E fluorescence intensity and macrophage count per field between paired tumour and tumour adjacent tissues. (E) CLEC4E fluorescence comparison between tumours from melanoma patients in stage I/II versus stage III/IV. (F) Overall survival analysis of patients with CLEC4E high and low expressions. Median CLEC4E fluorescence level was determined as the cutoff. (G) Overall survival analysis of patients with high CD68^+^ infiltration and high CLEC4E expression and patients with low CD68^+^ infiltration and low CLEC4E expression. Median CLEC4E fluorescence level and median CD68^+^ infiltration level were determined as the cutoffs.

Peritoneal macrophages were induced to TAMs with B16 conditioned medium, with M2 polarisation confirmed by marker expression (Figure S1a). RNA sequencing revealed that CLEC4E was significantly upregulated and highly expressed in TAMs compared to M0 macrophages (Figure 1A,B). We next evaluated the distribution of CLEC4E expression in paired tumour and tumour adjacent tissues from melanoma patients. As a result, there was a strong co‐localisation of CLEC4E with the macrophage marker CD68 in the tumour. In comparison, tumour adjacent tissue exhibited diminished expression of CLEC4E and less frequent macrophages (Figure 1C,D). To further validate the expression pattern of CLEC4E, we analysed a skin carcinoma cohort from the TCGA database. CLEC4E expression was significantly elevated in tumours compared with normal tissues, and showed a positive correlation with macrophage markers (CD14 and CD68), but not tumour cell markers (S100B and MIA) (Figure S1b,c). These results suggest that CLEC4E is specifically enriched in macrophages infiltrating the tumour microenvironment.

We next investigated the clinical relevance of CLEC4E expression in melanoma patients. Tumour tissues from 32 melanoma patients were analysed for both CLEC4E levels and TAM abundance. Patients diagnosed with stage III/IV diseases exhibited significantly higher CLEC4E expression compared to those with stage I/II tumours (Figure 1E). Importantly, survival analysis showed higher CLEC4E expression was associated with unfavourable prognosis (Figure 1F). Similarly, patients with more CLEC4E^+^ TAMs infiltration had poor clinical outcomes (Figure S1d). Furthermore, patients with more CD68^+^ TAMs and high CLEC4E expression had worse clinical outcomes compared to those with less TAMs and low CLEC4E expression (Figures 1G and S1e).

### 3.2 Macrophage‐expressing CLEC4E promotes tumour progression

To explore the potential role of CLEC4E in TAMs, we generated myeloid‐specific CLEC4E knockout mice by crossing Clec4e^flox^/^flox^ mice with Lyz2‐cre mice (referred to as Lyz2 ΔCLEC4E). The detailed strategy is shown in Figure S2a,b. Clec4e^flox/flox^ Lyz2‐cre**^+^** and Clec4e^flox/flox^ Lyz2‐cre**^−^** mice were used as knockout (Lyz2 ΔCLEC4E) and control groups, respectively. The efficient deletion of CLEC4E was confirmed by genotyping PCR and significantly reduced CLEC4E expression in macrophages from knockout mice (Figure S2c,d). No significant difference in body weight was observed between knockout and control mice (Figure S2e).

**FIGURE 2:**
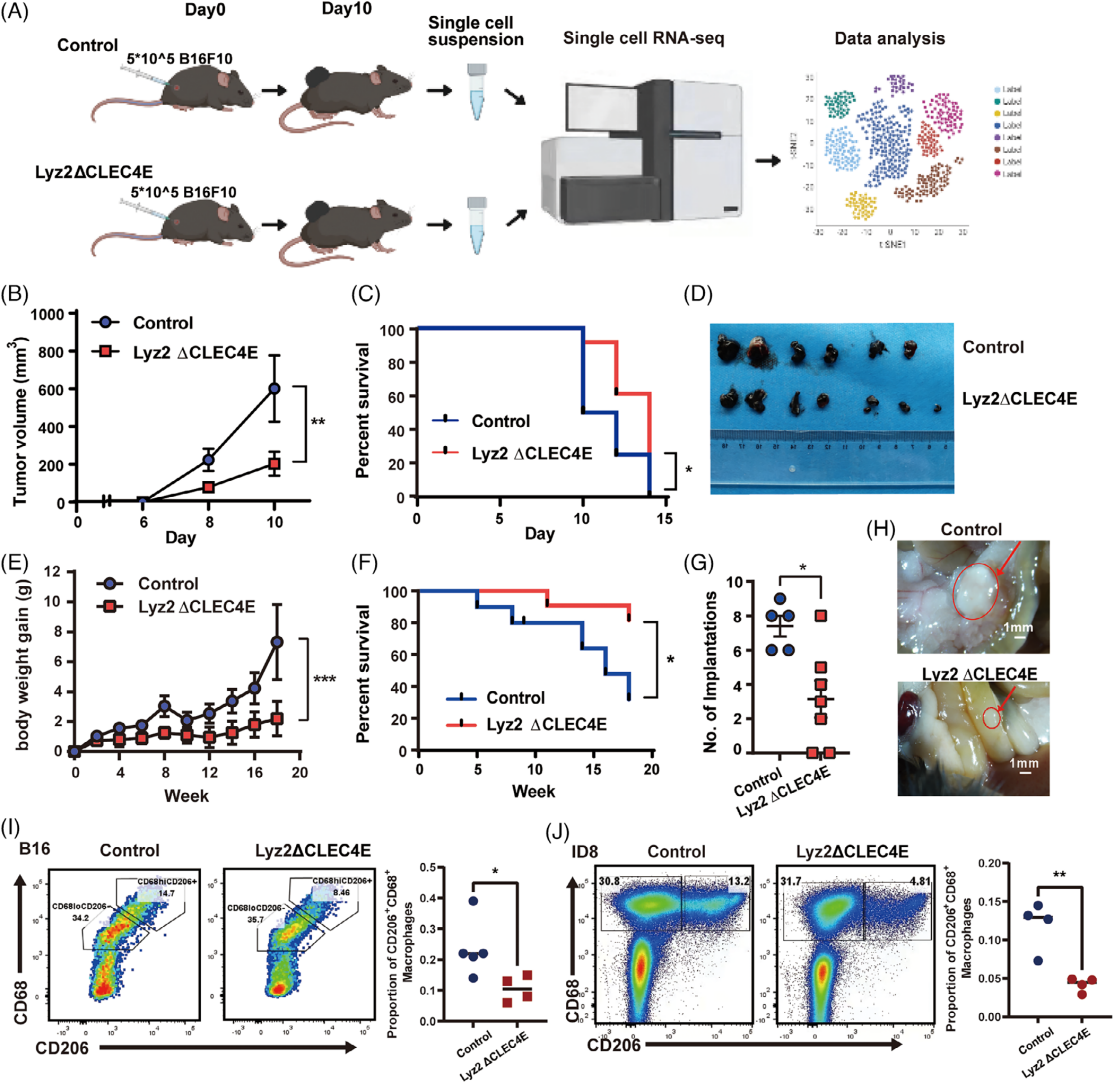
C‐type lectin domain family 4 member E (CLEC4E) expression on tumour‐associated macrophage (TAM) promotes tumour growth in mouse models. (A) Workflow of melanoma mouse model of CLEC4E conditional knockout mice. (B) Tumour growth curve of B16F10 melanoma model. (C) Survival analysis of B16F10 melanoma model. Tumour volume exceeding 500 mm^3^ was considered as the endpoint. (D) Pictures of melanoma tissues from CLEC4E knockout and control groups. (E) Body weight gain since intraperitoneal injection of ID8 cells in ovarian cancer model. (F) Survival analysis of ID8 ovarian cancer model. Body weight gain exceeding 4 g was considered as the endpoint. (G) Comparison of celiac tumour implantations in CLEC4E knockout and control groups. (H) Representative pictures of intestinal implantations. (I) Flow cytometry analysis of CD206 and CD68 from melanoma tissues at day 10. (J) Flow cytometry analysis of CD206 and CD68 from ovarian model ascites at week 8.

Lyz2 ΔCLEC4E and control mice were then subjected to subcutaneous melanoma model, and tumour growth was observed and tissues were harvested for scRNA‐seq (Figure 2A). Tumour growth was significantly suppressed in Lyz2 ΔCLEC4E mice, and the knockout animals exhibited improved survival compared to controls (Figure 2B‒2D). To further validate these findings, an ovarian cancer peritoneal implantation model was established via intraperitoneal injection with ID8 cells. Ascites formation, reflected by increased body weight, was significantly reduced in Lyz2 ΔCLEC4E mice, which also showed prolonged survival (Figure 2E,F). At week 18, all mice were sacrificed and examined for tumour burden. Lyz2 ΔCLEC4E mice were found to have fewer and smaller peritoneal tumour nodules than control animals (Figure 2G,H). In addition, flow cytometry of tumours from both groups revealed that CLEC4E knockout decreased the frequency of CD206^+^ TAMs, indicative of a diminished M2‐like pro‐tumour macrophage polarisation (Figures 2I,J and S2f).

### 3.3 CLEC4E deletion reprograms TAM towards an anti‐tumour phenotype

To investigate the mechanism by which CLEC4E regulates tumour growth, we performed scRNA‐seq on melanoma tissue from our mouse model. The major cell populations, including B16 tumour cells, endothelial cells, fibroblasts, myeloid cells and T cells were identified (Figure S3a). The characteristic markers and their distribution are illustrated in Figure S3b,c. Notably, the proportion of tumour cells significantly decreased with CLEC4E deletion (Figure S3d).

**FIGURE 3:**
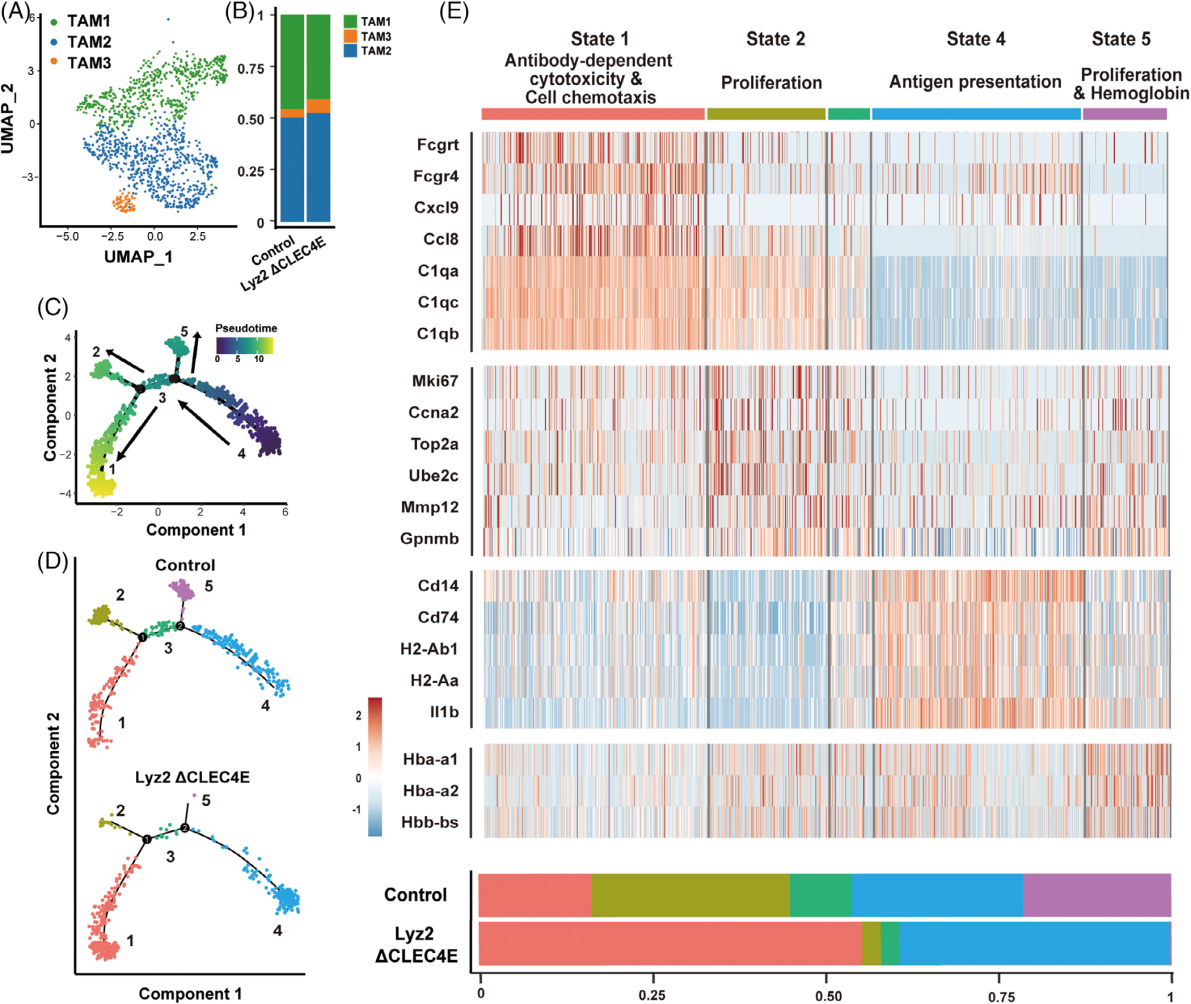
Single‐cell RNA sequencing analysis of macrophages from melanoma tissues of C‐type lectin domain family 4 member E (CLEC4E) knockout and control mice. (A) Uniform manifold approximation and projection (UMAP) plot of total macrophages. (B) Bar chart showing proportions of macrophage clusters in CLEC4E knockout and control mice. (C) Pseudotime trajectory of macrophages. Cells were divided into five states. (D) Pseudotime trajectories and bar chart showing macrophage distributions of CLEC4E knockout and control mice. (E) Heatmap showing gene markers of each state and the proportion comparison between knockout and control mice. CLEC4E knockout mice had enriched macrophages in states 1 and 4, and decreased macrophages in states 2 and 5 compared to control mice.

We next focused on the myeloid cluster to dissect the role of macrophages. As shown in Figure S3f,g, macrophages constituted the most majority of the myeloid population, with dendritic cells made up the remainder. Comparison of Clec4e expression revealed minimal detection in dendritic cells (Figure S3i). Macrophages were further subdivided into three subclusters (TAM1, TMA2, TAM3), shown in Figures 3A and S3e. However, no differences were observed between Lyz2 ΔCLEC4E and control groups in the proportion of these subclusters (Figure 3B).

Therefore, we performed pseudotime analysis to reconstruct the developmental trajectory of TAMs, categorising cells into five states (Figure 3C). Pseudotime trajectories help reconstructing the dynamic development and identify differentiation stages of a cell population. States 1 and 2 corresponded primarily to Mac2 and Mac3 clusters, while states 4 and 5 aligned with Mac1 cluster (Figure S3h). The distribution of cells from Lyz2 ΔCLEC4E and control groups differed along this trajectory. Specifically, cells in states 2 and 5 decreased, while those of states 1 and 4 expanded (Figure 3D).

Representative gene markers for each state are shown in Figure 3E. State 1 was characterised by expressing chemokines and antibody‐dependent receptors mediating anti‐tumour responses, suggesting a pro‐inflammatory phenotype. State 2 featured gene markers associated with cell proliferation, representing a proliferating TAM subset. State 4 highly expressed antigen presentation‐related genes, also indicating anti‐tumour functionality. State 5 expressed hemoglobin‐related genes as well as cell proliferation markers similar to state 2 (Figure 3E). To conclude, CLEC4E deletion suppressed the proportion of proliferating TAMs and expanded the macrophages populations with antigen presentation capacity and anti‐tumour response.

As shown in Figure S3h, macrophages in states 1 and 2 were distributed within overlapping clusters, while State 4 and State 5 exhibit substantial co‐localisation. We further performed pathway enrichment analysis on state 1 versus state 2 and state 4 versus state 5. The two comparison yielded similar pathways. Cytokine‐related response, antigen processing and presentation, and defense response were enriched in states 1 and 4—the two subsets that were expanded in Lyz2 ΔCLEC4E mice (Figure S3j).

To validate the result from scRNA‐seq, we obtained peritoneal macrophages from Lyz2 ΔCLEC4E and control mice, followed by RT‐PCR analysis. Expressions of chemokines (CXCL9 and CCL8), antigen receptors (FCGR4 and FCGRT), the pro‐inflammatory cytokine (IL1B) and antigen presentation molecules (CD74, H2AA and H2AB1) were upregulated in CLEC4E knockout macrophages compared with controls, confirming the differential expression patterns observed in the sequencing data (Figure S3k).

### 3.4 TAM proliferation is inhibited in the absence of CLEC4E through ERK/MAPK pathway

**FIGURE 4:**
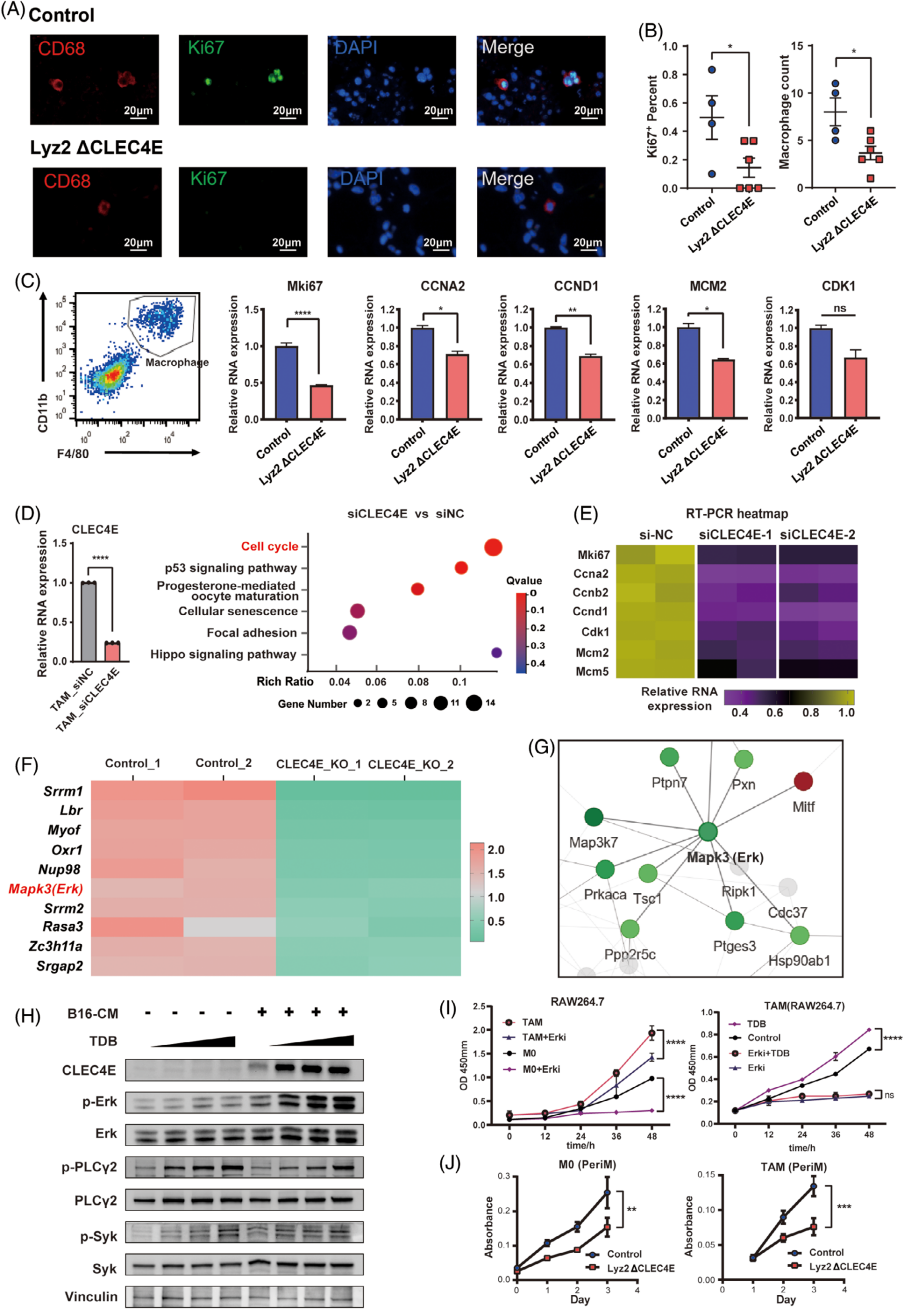
C‐type lectin domain family 4 member E (CLEC4E) deletion suppresses tumour‐associated macrophage (TAM) proliferation and abundance in tumour microenvironment (TME). (A) Immunofluorescence of CD68 and Ki67 with B16F10 melanoma tumours from CLEC4E knockout and control mice. (B) Column charts comparing Ki67^+^ cells in TAM and macrophage count between control and CLEC4E knockout groups. (C) Macrophage sorting chart and qRT‐PCR comparing proliferation markers in macrophages from control and CLEC4E knockout mice. (D) qRT‐PCR of CLEC4E silencing efficiency and Kyoto Encyclopedia of Genes and Genomes (KEGG) enrichment analysis with CLEC4E silencing in TAM. (E) RT‐PCR verification of proliferation gene markers enriched in KEGG analysis. (F) Top 10 differentially expressed phosphorylated proteins between CLEC4E knockout and control macrophages. (G) Protein‒protein interaction (PPI) analysis of differentially expressed phosphorylated proteins in CLEC4E knockout versus control macrophages. (H) Western blot of PLC‐γ2, Syk and Erk phosphorylation with CLEC4E ligation in macrophages. (Trehalose‐6,6‐dibehenate) TDB concentrations were 0, 10, 25 and 50 µg/mL sequentially. (I) Cell counting kit‐8 assay of M0 and TAMs differentiated from RAW264.7 with TDB or Erk inhibitor (Erki). (j) Cell counting kit‐8 assay of peritoneal macrophages from CLEC4E knockout and control mice.

To confirm that CLEC4E knockout suppresses TAM proliferation, we evaluated Ki67 expression in macrophages within tumour tissues. Immunofluorescence analysis showed a reduced proportion of Ki67^+^ macrophages and a lower macrophage count per field in Lyz2 ΔCLEC4E group compared to controls (Figure 4A,B). Next, we sorted F4/80^+^CD11b^+^ macrophages from tumours of both groups and performed RT‐PCR to assess proliferation markers. Consistent with the immunofluorescence results, the expressions of MKI67, CCNA2, CCND1, MCM2 and CDK1 were inhibited in the Lyz2 ΔCLEC4E group (Figure 4C).

To further validate these findings, we performed ex vivo silencing of CLEC4E in BMDMs using siRNA. Total RNA was subjected to sequencing. KEGG pathway analysis of DEGs showed that cell cycle pathway was the most significantly enriched in CLEC4E‐silenced macrophages versus controls (Figure 4D). RT‐PCR further verified the downregulation of key cell cycle‐related genes identified in the sequencing data, including MKI67, CCNA2, CCNB2, CCND1, CDK1, MCM2 and MCM5 (Figure 4E).

To explore the mechanism by which CLEC4E regulates cell proliferation, BMDM from Lyz2 ΔCLEC4E and control mice were subjected to proteomic and phosphoproteomic sequencing. As a result, the phosphorylation of Mapk3 (Erk1) was significantly decreased in CLEC4E knockout macrophages (Figure 4F). Furthermore, the protein‒protein interaction analysis showed that Mapk3 (Erk1) was an essential protein whose phosphorylation was notably suppressed in CLEC4E knockout group (Figure 4G).

To verify the involvement of the Erk pathway, macrophages were stimulated with the CLEC4E ligand TDB to activate CLEC4E signalling. Phosphorylation levels of Erk, PLC‐γ2 and Syk were subsequently assessed. Both M0 macrophages and TAMs exhibited enhanced phosphorylation of Erk, Syk and PLC‐γ2 in response to increasing concentrations of TDB (Figure 4H). This suggested that Erk pathway acts as a downstream signalling target of CLEC4E in TAMs. Furthermore, CCK‐8 assays performed in RAW264.7 cells demonstrated TDB‐induced proliferation was effectively suppressed by Erk inhibitor treatment (Figure 4I). We then isolated macrophages from Lyz2 ΔCLEC4E and control mice. The results showed that proliferation was significantly reduced in macrophages from CLEC4E knockout mice compared to those from control mice, under both M0 and TAM‐polarising conditions (Figure 4J).

### 3.5 CLEC4E knockout strengthens macrophage‒T cell interaction and promotes T‐cell cytotoxicity

**FIGURE 5:**
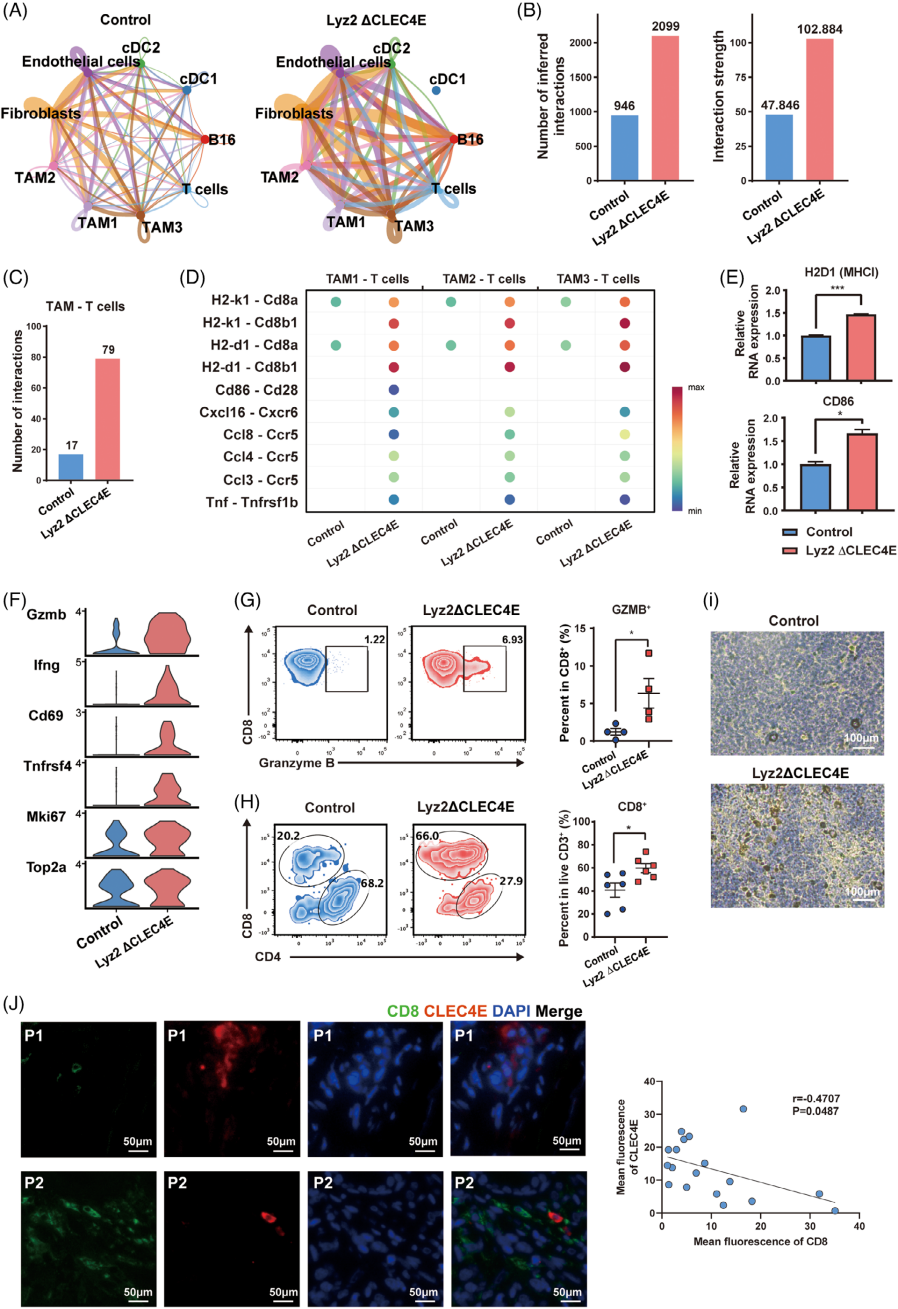
C‐type lectin domain family 4 member E (CLEC4E) knockout strengthens macrophage‒T cell interaction and T‐cell cytotoxicity. (A) Interactions of all cell clusters in control and CLEC4E knockout mice. (B) Bar charts showing the number and strength of total interactions. (C) Bar chart of interaction numbers of tumour‐associated macrophage (TAM)‒T cells. (D) Selective ligand‒receptor pair expressions between TAM and T cells. (E) Bar chart of RT‐PCR with sorted macrophages from mouse melanoma tissues. (F) Expressions of selective genes in T‐cell population. (G) Flow cytometry of granzyme B and CD8 in ovarian cancer ascites at week 18. (H) Flow cytometry of CD4 and CD8 in melanoma tissues at day 10. (I) Immunohistochemistry of granzyme B with intestinal implantation of ovarian cancer. (J) Immunofluorescence images of melanoma tumour tissues from two patients and the correlation between the area of mean fluorescence of CLEC4E and CD8 of 18 patients.

To investigate the impact of CLEC4E knockout on the tumour microenvironment, we performed the cell‒cell interaction analysis on tumours from both Lyz2 ΔCLEC4E and control mice. Overall, the cellular interactions were enhanced in Lyz2 ΔCLEC4E tumours (Figure 5A). Specifically, the total number of inferred interactions and their overall strength were significantly increased in the knockout group (Figure 5B). Interactions between macrophages and T cells, in particular, increased fivefold upon CLEC4E knockout (Figure 5C). Analysis of ligand‒receptor pair revealed strengthened interactions between macrophage and T cells, including H2‐K1/H2‐D1 (MHCI)‒CD8 and CD86‒CD28 pairs, which are critical for signals 1 and 2 in T‐cell activation process, respectively (Figure 5D). Additionally, the expressions of T‐cell‐attracting chemokines were upregulated in the knockout group (Figure 5D). RT‐PCR with sorted TAMs further validated the increased expression of MHCI and CD86 in CLEC4E knockout mice, consistent with the enhanced interaction patterns observed (Figure 5E).

Next, we investigated how T‐cell population were altered following macrophage‐specific CLEC4E deletion. Analysis of T‐cell activation and proliferation markers showed increased expressions of Gzmb, IFN‐γ, Tnfrsf4, Mki67 and Top2a, indicating enhanced T‐cell activation and functional capacity (Figure 5F). Furthermore, flow cytometry with tumour tissues demonstrated an elevated proportion of granzyme B‐expressing CD8^+^ T cells (Figure 5G), which was supported by consistent granzyme B immunohistochemistry staining results (Figure 5I). Since scRNA‐seq could not distinguish CD4^+^ and CD8^+^ T‐cell clusters, we used flow cytometry analysis to evaluate T‐cell subsets, which showed an increased CD8^+^ T‐cell infiltration (Figure 5H). Lastly, we explored the relationship between CLEC4E expression and CD8^+^ T cells abundance in patient samples. Immunofluorescence analysis demonstrated an inverse correlation: tumours with higher CLEC4E expression contained fewer CD8^+^ cells, and vice versa (Figure 5J). Collectively, these data revealed that CLEC4E knockout in macrophage enhances macrophage‒T cell interactions and promotes CD8^+^ T‐cell cytotoxicity within tumour microenvironment.

### 3.6 BET inhibitor is a strong candidate for CLEC4E inhibition

**FIGURE 6:**
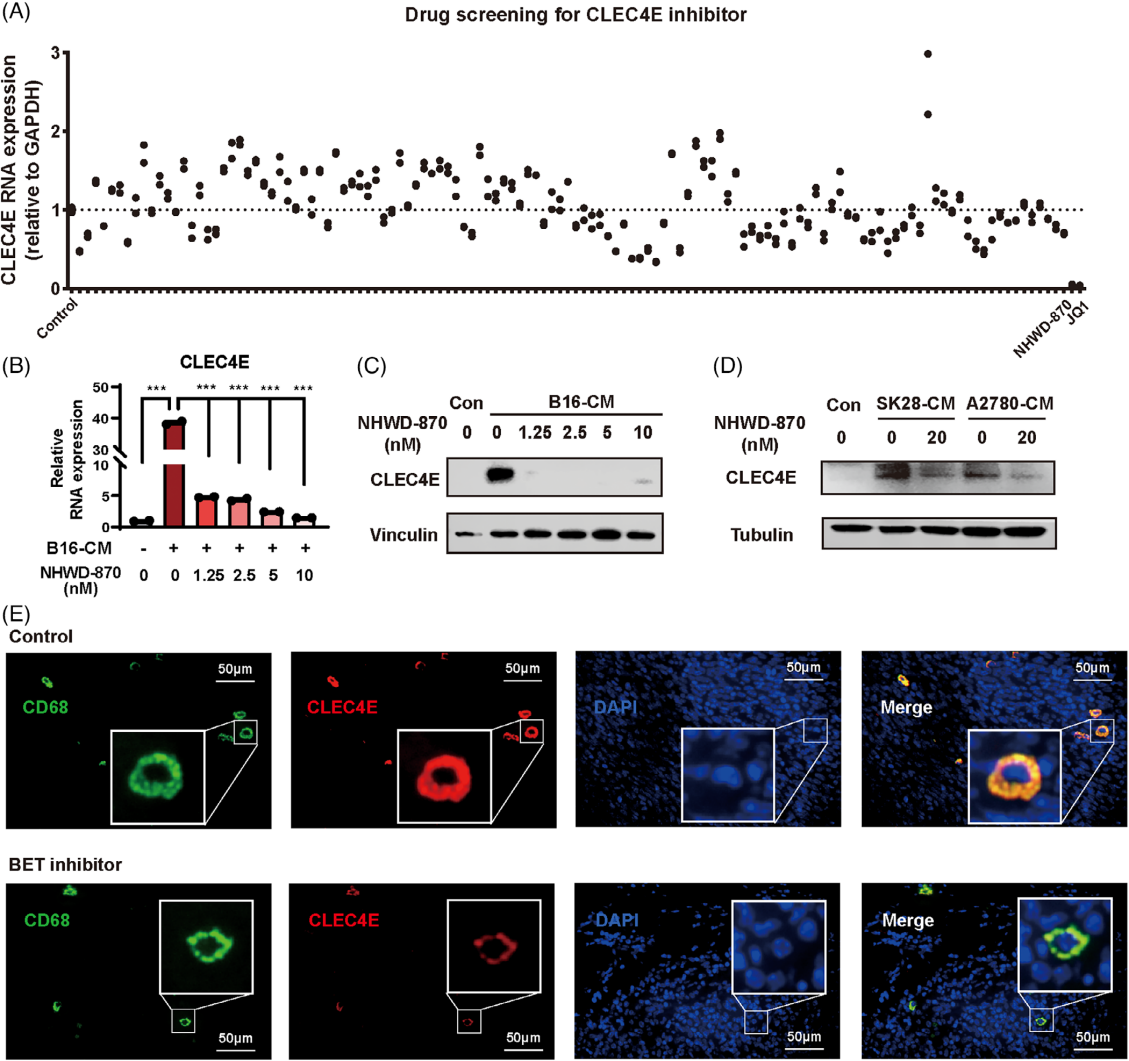
BET inhibitor strongly suppresses C‐type lectin domain family 4 member E (CLEC4E) expression on tumour‐associated macrophage (TAM). (A) Screening of 132 drugs for CLEC4E inhibition in RAW264.7 TAM induced by B16‐CM. (B) RT‐PCR of CLEC4E expression on BMDM with B16‐CM and NHWD‐870 treatment. (C) Western blot of CLEC4E expression on BMDM with B16‐CM and NHWD‐870 treatment. (D) Western blot of CLEC4E expression on THP‐1 TAM induced with SK28‐CM or A2780‐CM. (E) Immunofluorescence of Yumm1.7 melanoma tissues with BET inhibitor NHWD‐870 treatment.

Given the unfavourable role of macrophage CLEC4E in the TME, we conducted a drug screen to identify CLEC4E inhibitor. From an FDA‐approved drug library, we prioritised compounds targeting immunology/infection pathways—consistent with the immunomodulatory function of CLEC4E—as well as epigenetic agents, due to the importance of epigenetic regulation in cancer progression. In total, 132 drugs were applied to B16‐conditioned medium induced RAW264.7 macrophages, and CLEC4E expression was examined. Among these, BET inhibitors JQ1 and NHWD‐870 showed the most pronounced decrease of CLEC4E levels (Figure 6A). We further validated this effect using BMDMs. CLEC4E was upregulated upon B16‐CM stimulation and significantly suppressed by low dose of BET inhibitor treatment (Figure 6B). The protein level was also assessed, which showed the consistent result (Figure 6C).

In addition, human monocyte THP‐1 cells were induced to TAM using conditioned medium melanoma and ovarian cancer cells. CLEC4E was enriched in TAMs and suppressed by BET inhibitor in both types of TAM (Figure 6D). The regulation was then evaluated in the animal model. The subcutaneous melanoma model with Yumm1.7 cells was constructed, with NHWD‐870 or vehicle administered at 1 mg/kg. Detailed method can be found in our previous publication.^22^ Immunostaining of tumour tissues revealed strong co‐localisation of CLEC4E with the macrophage marker CD68 in both groups. However, CLEC4E fluorescence intensity was markedly reduced in the BET inhibitor‐treated group (Figure 6E).

### 3.7 CLEC4E is suppressed by BET inhibitor via BRD4/CEBPB regulation

**FIGURE 7:**
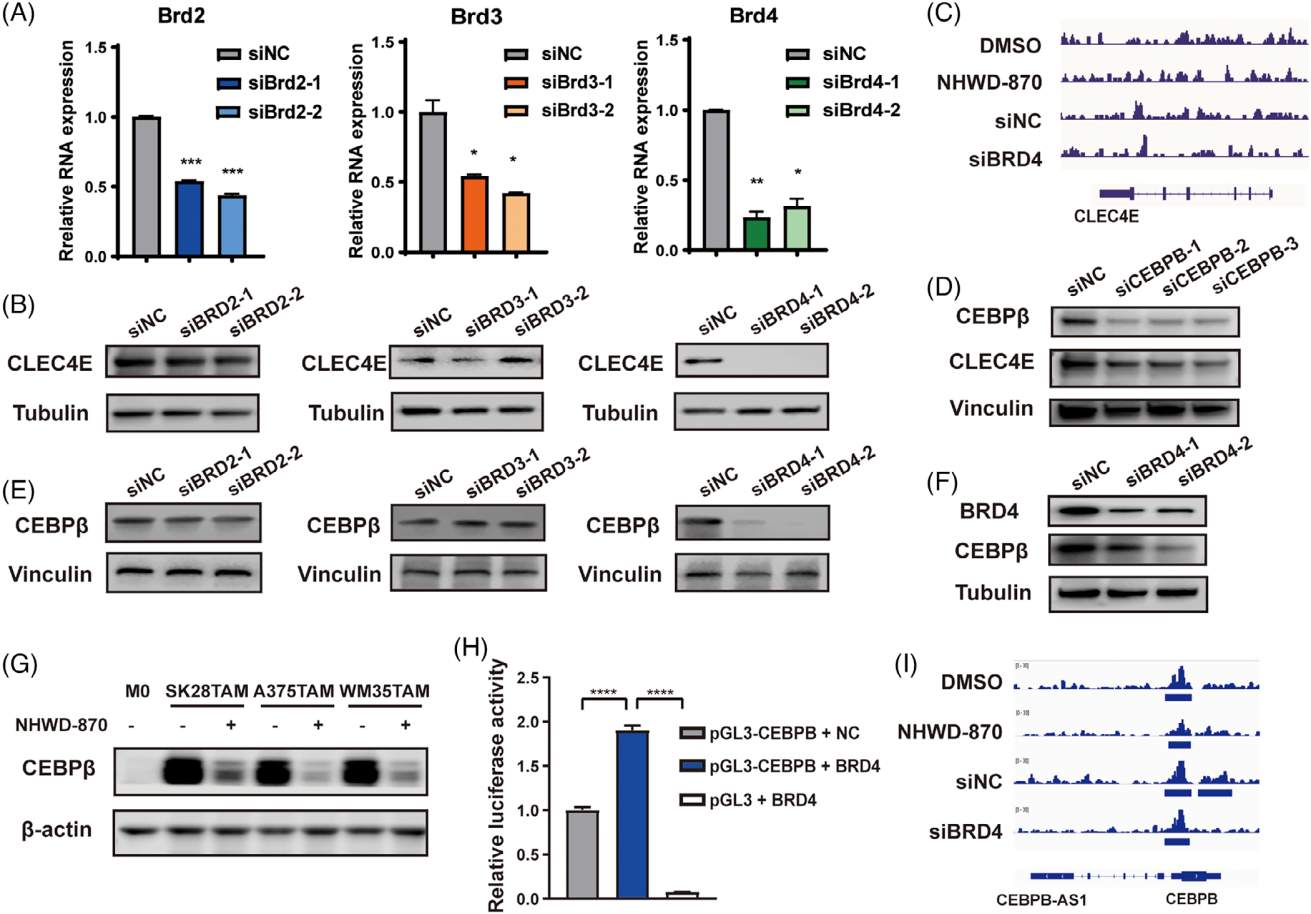
BET inhibitor downregulates C‐type lectin domain family 4 member E (CLEC4E) by targeting on BRD4/CEBPβ. (A) RT‐PCR showing the efficiency of RNA silencing of BRD2, BRD3 and BRD4 on BMDM‐TAM. (B) Western blot of CLEC4E with BRD2/3/4 silenced in BMDM‐TAM. (C) CHIP sequencing (CHIP‐seq) of A375 showing no BRD4 binding on CLEC4E. (D) Western blot of CLEC4E with CEBPβ silenced in BMDM‐TAM. (E) Western blot of CEBPβ with BRD2/3/4 silenced in BMDM‐TAM. (F) Western blot of CEBPβ with BRD4 silenced in THP‐1 tumour‐associated macrophage (TAM). (G) Western blot of CEBPβ with BET inhibitor NHWD‐870 (20 nM) treatment in THP‐1 TAM induced by indicated tumour conditioned medium. (H) Luciferase assay with 293T cells transfected with pGL3‐CEBPB or pLG3‐basic and BRD4 or NC plasmids. (I) CHIP‐seq of A375 showing BRD4 binding on the promoter of CEBPB.

To understand the mechanism of CLEC4E downregulation by BET inhibitor, we first silenced BRD2, BRD3 and BRD4 individually in BMDMs, as they are the primary targets of BET inhibitor. The efficiency of knockdown by each siRNA was confirmed (Figure 7A). Subsequent analysis revealed that CLEC4E expression remained unchanged following BRD2 or BRD3 knockdown but was significantly reduced upon BRD4 depletion (Figure 7B). We then performed ChIP‐seq to assess whether BRD4 protein binds directly to the CLEC4E genomic locus. However, no significant BRD4 binding peaks were detected within any region of the CLEC4E gene, suggesting that BRD4 may not regulate CLEC4E directly (Figure 7C).

In review of previous literatures, we found that CLEC4E expression is regulated dependent on the transcription factor CCAAT/enhancer binding protein (CEBPβ).^34^, ^35^ We first confirmed this regulatory relationship by silencing CEBPB in THP‐1 cells, which resulted in downregulation of CLEC4E (Figure 7D). We next explored the regulation of BET inhibitor and BRD4 on CEBPB. Knockdown of BRD4—but not BRD2 or BRD3—in BMDMs led to a significant decrease in CEBPβ, consistent with the expression pattern of CLEC4E (Figure 7E). This effect was corroborated in human macrophages: BRD4 knockdown in THP‐1 cells also reduced CEBPβ levels (Figure 7F). Furthermore, BMDM was treated with conditioned medium from different types of tumour cells to induce TAM, with or without BET inhibitor. It was found that BET inhibitor significantly suppressed the CEBPβ expression in TAMs (Figure 7G).

To determine whether BRD4 directly regulates CEBPB transcription, we performed a dual‐luciferase reporter assay. Overexpression of BRD4 significantly enhanced activity of the CEBPB promoter (Figure 7H). We then observed binding of BRD4 on the promoter region of CEBPB by ChIP‐seq, and this binding was diminished upon BET inhibitor treatment or BRD4 silencing, indicating direct transcriptional regulation (Figure 7I). Taken together, these results demonstrate that BET inhibitors suppress CLEC4E expression by targeting BRD4, which directly regulates CEBPβ—a known upstream modulator of CLEC4E.

## 4 DISCUSSION

Our study identifies a pro‐tumour role for macrophage‐expressed CLEC4E in melanoma and ovarian cancer. This broadened our understanding of its function in the context of tumour. Previous research on CLEC4E primarily focused on the pulmonary and gastrointestinal systems. It was reported that inhibitory macrophages lost their immunosuppressive effects following the deletion of CLEC4E in pancreatic ductal adenocarcinoma (PDA) models.^36^ High expression of CLEC4E was associated with unfavourable survival in non‐small cell lung cancer patients.^20^ Moreover, the CLEC4 family has been implicated in the development of hepatocellular carcinoma.^37^ CLEC4E is significantly upregulated in GC, a phenomenon closely linked to a poor prognosis.^38^ Knockdown of CLEC4E in macrophages is found to result in a decrease in phagocytic capacity.^39^ Consistent with these results, our study suggest an unfavourable role of CLEC4E on melanoma and ovarian cancer, confirming it as a potential therapeutic target.

In this study, we generated a myeloid‐specific conditional knockout mouse model to investigate the role of CLEC4E in tumours. This model rules out the effect of CLEC4E on other cell types, which makes it superior to other studies where the complete knockout model was used.^19^, ^40^, ^41^, ^42^ Using the advantage of scRNA‐seq, we have a comprehensive understanding of how CLEC4E mediates the immunosuppressive function of TAM. We are the first to illustrate that CLEC4E contributes to TAM proliferation. As the abundance of TAM significantly correlates with patient survival, TAM proliferation could be an immunosuppressive factor.^3^, ^4^, ^5^ Proteomics sequencing further identifies the Mapk/Erk pathway as a downstream molecule of CLEC4E. A previous study showed that TDB, a ligand of CLEC4E, activated PLC‐γ1/PKC/ERK signalling, but independent of CLEC4E.^43^ This might be due to the use of a different cell line, microglia, in their study, while BMDM was used in ours, making a difference in the context and background in cell signalling. However, in this article, we did not conduct adoptive transfer experiment for further validation, which is a main limitation of our research. In general, this study reveals novel mechanism of CLEC4E in the tumour and updates our knowledge on the immunosuppression of TAM.

Furthermore, our study clarifies the impact of CLEC4E on the tumour immune microenvironment. Deletion of CLEC4E is shown to strengthen the interaction between macrophage and T cell. Previous studies have discussed the association between CLEC4E and CD4^+^ T cells. For example, CLEC4E signalling in dendritic cells regulates IL‐17 and IL‐22 production in CD4^+^ T cells and reinforces intestinal immune barrier.^44^ CD40 signalling in B cells induces T‐cell proliferation through a CLEC4E‐dependent pathway.^42^ The only research investigating CLEC4E and T cells in tumours shows that complete knockout of CLEC4E increases IFN‐γ production by both CD4^+^ and CD8^+^ T cells in PDA.^36^ This aligns consistent with our findings in terms that cytotoxicity of CD8^+^ T cells is enhanced with CLEC4E deletion. Furthermore, while CLEC4E interacts with CD4^+^ T cells in inflammatory conditions, it shows more robust effect on CD8^+^ T cells in our tumour model. Not only the ligand‒receptor changes emphasises the enhanced MHCI‒CD8 correlation, but also CD8^+^ T cells frequency and its granzyme B expression are increased with CLEC4E deletion. Therefore, our study helps with better understanding of how CLEC4E interacts with other cell populations in TME and exerts immunosuppressive role.

The BET inhibitor, NHWD‐870, is shown to strongly inhibit CLEC4E by targeting the transcription factor C/EBPβ in our study. The BET inhibitor is a type of newly developed anti‐cancer agents, of which molecular mechanism is still in explore. The direct target genes of BRD4 are mainly transcription factors included MYC, BCL2 and IL7R.^45^, ^46^, ^47^, ^48^ We report for the first time that BRD4 directly binds to the promoter of C/EBPβ in macrophages. C/EBPβ is typically associated with endoplasmic reticulum stress (ER stress) and inflammatory conditions, and it also contributes to cancer progression.^49^, ^50^, ^51^, ^52^, ^53^, ^54^, ^55^ By suppressing C/EBPβ expression, the BET inhibitor regulates CLEC4E, and might also mediate other immunoregulatory genes, which needs further investigation and potentially reveals more mechanisms of BET inhibitor.

In conclusion, CLEC4E^+^ TAM exerts immunosuppressive role by promoting TAM proliferation, repressing anti‐tumour responses and inactivating T‐cell cytotoxicity. BET inhibitor serves as a potential therapeutic approach for CLEC4E inhibitor by targeting BRD4/CEBPβ.

## 5 CONCLUSION

In summary, CLEC4E expression on TAMs promotes an immunosuppressive tumour microenvironment and drives tumour progression by facilitating TAM proliferation and impairing T‐cell function. The BET inhibitor potently suppressed CLEC4E expression in TAMs by targeting BRD4/CEBPβ axis, thereby restoring macrophage anti‐tumour activity and enhancing T‐cell‐mediated cytotoxicity, ultimately leading to suppressed tumour growth.

## AUTHOR CONTRIBUTIONS

Mengting Liao designed and performed the experiment, analysed and interpretated the data, and drafted the manuscript. Kexin Long performed the experiment, analysed the data and drafted the manuscript. Liang Dong analysed and interpretated the data. Zhuo Li, Wenhua Wang, Yangyi Zhang and Rui Hu performed the experiment. Juan Su contributed to acquisition of data. Wu Zhu, Xiang Chen and Mingzhu Yin contributed to conception and design of the study. All the authors revised the manuscript and gave final approval to the version submitted for publication.

## CONFLICT OF INTEREST STATEMENT

The authors declare that they have no known competing financial interests or personal relationships that could have appeared to influence the work reported in this paper.

## ETHICS STATEMENT

This study was approved by the institutional research ethics boards of Xiangya Hospital, Central South University, China (no. 202308636). Written informed consent was obtained from all participants. All protocols were approved by the Ethics Committee on Laboratory Animals of Xiangya Medicine School of Central South University (no. CSU‐2022‐01‐0472).

## CONSENT FOR PUBLICATION

The consent from all authors was obtained.

## Supporting information



Supporting Information

Supporting Information

Supporting Information

## Data Availability

The datasets used and analysed during the current study are available from the corresponding author upon reasonable request.

## References

[1] Ngambenjawong C , Gustafson HH , Pun SH . Progress in tumor‐associated macrophage (TAM)‐targeted therapeutics. Adv Drug Deliv Rev. 2017;114:206‐221. 28449873 10.1016/j.addr.2017.04.010 PMC5581987

[2] Ruffell B , Coussens LM . Macrophages and therapeutic resistance in cancer. Cancer Cell. 2015;27:462‐472. 25858805 10.1016/j.ccell.2015.02.015 PMC4400235

[3] Hwang I , Kim JW , Ylaya K , et al. Tumor‐associated macrophage, angiogenesis and lymphangiogenesis markers predict prognosis of non‐small cell lung cancer patients. J Transl Med. 2020;18:443. 33228719 10.1186/s12967-020-02618-z PMC7686699

[4] Mehta AK , Kadel S , Townsend MG , Oliwa M , Guerriero JL . Macrophage biology and mechanisms of immune suppression in breast cancer. Front Immunol. 2021;12:643771. 33968034 10.3389/fimmu.2021.643771 PMC8102870

[5] Cheng K , Cai N , Zhu J , Yang X , Liang H , Zhang W . Tumor‐associated macrophages in liver cancer: from mechanisms to therapy. Cancer Commun (Lond). 2022;42:1112‐1140. 36069342 10.1002/cac2.12345 PMC9648394

[6] Xiang X , Wang J , Lu D , Xu X . Targeting tumor‐associated macrophages to synergize tumor immunotherapy. Signal Transduct Target Ther. 2021;6:75. 33619259 10.1038/s41392-021-00484-9 PMC7900181

[7] Lopez‐Yrigoyen M , Cassetta L , Pollard JW . Macrophage targeting in cancer. Ann N Y Acad Sci. 2021;1499:18‐41. 32445205 10.1111/nyas.14377

[8] Advani R , Flinn I , Popplewell L , et al. CD47 blockade by Hu5F9‐G4 and rituximab in non‐Hodgkin's lymphoma. N Engl J Med. 2018;379:1711‐1721. 30380386 10.1056/NEJMoa1807315 PMC8058634

[9] Razak AR , Cleary JM , Moreno V , et al. Safety and efficacy of AMG 820, an anti‐colony‐stimulating factor 1 receptor antibody, in combination with pembrolizumab in adults with advanced solid tumors. J Immunother Cancer. 2020;8:e001006. 33046621 10.1136/jitc-2020-001006 PMC7552843

[10] Machiels J‐P , Gomez‐Roca C , Michot J‐M , et al. Phase Ib study of anti‐CSF‐1R antibody emactuzumab in combination with CD40 agonist selicrelumab in advanced solid tumor patients. J Immunother Cancer. 2020;8:e001153. 33097612 10.1136/jitc-2020-001153 PMC7590375

[11] Tap WD , Gelderblom H , Palmerini E , et al. Pexidartinib versus placebo for advanced tenosynovial giant cell tumour (ENLIVEN): a randomised phase 3 trial. Lancet. 2019;394:478‐487. 31229240 10.1016/S0140-6736(19)30764-0 PMC6860022

[12] Lu X , Nagata M , Yamasaki S . Mincle: 20 years of a versatile sensor of insults. Int Immunol. 2018;30:233‐239. 29726997 10.1093/intimm/dxy028

[13] Patin EC , Orr SJ , Schaible UE . Macrophage inducible C‐type lectin as a multifunctional player in immunity. Front Immunol. 2017;8:861. 28791019 10.3389/fimmu.2017.00861 PMC5525440

[14] Fisher J , Card G , Liang Y , Trent B , Rosenzweig H , Soong L . Orientia tsutsugamushi selectively stimulates the C‐type lectin receptor Mincle and type 1‐skewed proinflammatory immune responses. PLoS Pathog. 2021;17:e1009782. 34320039 10.1371/journal.ppat.1009782 PMC8351992

[15] Inoue T . M1 macrophage triggered by Mincle leads to a deterioration of acute kidney injury. Kidney Int. 2017;91:526‐529. 28202166 10.1016/j.kint.2016.11.026

[16] Nagata M , Toyonaga K , Ishikawa E , et al. Helicobacter pylori metabolites exacerbate gastritis through C‐type lectin receptors. J Exp Med. 2021;218:e20200815. 32991669 10.1084/jem.20200815 PMC7527975

[17] N'Diaye M , Brauner S , Flytzani S , et al. C‐type lectin receptors Mcl and Mincle control development of multiple sclerosis‐like neuroinflammation. J Clin Invest. 2020;130:838‐852. 31725411 10.1172/JCI125857 PMC6994148

[18] Veltman D , Wu M , Pokreisz P , et al. Clec4e‐receptor signaling in myocardial repair after ischemia‒reperfusion injury. JACC Basic Transl Sci. 2021;6:631‐646. 34466750 10.1016/j.jacbts.2021.07.001 PMC8385568

[19] Li C , Xue VW , Wang Q‐M , et al. The Mincle/Syk/NF‐κB signaling circuit is essential for maintaining the protumoral activities of tumor‐associated macrophages. Cancer Immunol Res. 2020;8:1004‐1017. 32532809 10.1158/2326-6066.CIR-19-0782

[20] Zhou S , Sun Y , Chen T , et al. The landscape of the tumor microenvironment in skin cutaneous melanoma reveals a prognostic and immunotherapeutically relevant gene signature. Front Cell Dev Biol. 2021;9:739594. 34660598 10.3389/fcell.2021.739594 PMC8517264

[21] Jiang Q , Xiao D , Wang A , et al. CLEC4E upregulation in gastric cancer: a potential therapeutic target correlating with tumor‐associated macrophages. Heliyon. 2024;10:e27172. 38463883 10.1016/j.heliyon.2024.e27172 PMC10920739

[22] Yin M , Guo Y , Hu R , et al. Potent BRD4 inhibitor suppresses cancer cell‒macrophage interaction. Nat Commun. 2020;11:1833. 32286255 10.1038/s41467-020-15290-0 PMC7156724

[23] Wang Z‐Q , Zhang Z‐C , Wu Y‐Y , et al. Bromodomain and extraterminal (BET) proteins: biological functions, diseases, and targeted therapy. Signal Transduct Target Ther. 2023;8:420. 37926722 10.1038/s41392-023-01647-6 PMC10625992

[24] Faivre EJ , McDaniel KF , Albert DH , et al. Selective inhibition of the BD2 bromodomain of BET proteins in prostate cancer. Nature. 2020;578:306‐310. 31969702 10.1038/s41586-020-1930-8

[25] Hu R , Hou H , Li Y , et al. Combined BET and MEK inhibition synergistically suppresses melanoma by targeting YAP1. Theranostics. 2024;14:593‐607. 38169595 10.7150/thno.85437 PMC10758063

[26] Jiang Y , Wang G , Mu H , et al. Bromodomain inhibition attenuates the progression and sensitizes the chemosensitivity of osteosarcoma by repressing GP130/STAT3 signaling. Front Oncol. 2021;11:642134. 34168981 10.3389/fonc.2021.642134 PMC8219214

[27] Kulikowski E , Rakai BD , Wong NCW . Inhibitors of bromodomain and extra‐terminal proteins for treating multiple human diseases. Med Res Rev. 2021;41:223‐245. 32926459 10.1002/med.21730 PMC7756446

[28] Shu S , Lin CY , He HH , et al. Response and resistance to BET bromodomain inhibitors in triple‐negative breast cancer. Nature. 2016;529:413‐417. 26735014 10.1038/nature16508 PMC4854653

[29] Erkes DA , Field CO , Capparelli C , et al. The next‐generation BET inhibitor, PLX51107, delays melanoma growth in a CD8‐mediated manner. Pigment Cell Melanoma Res. 2019;32:687‐696. 31063649 10.1111/pcmr.12788 PMC6697571

[30] Kim E , Ten Hacken E , Sivina M , et al. The BET inhibitor GS‐5829 targets chronic lymphocytic leukemia cells and their supportive microenvironment. Leukemia. 2020;34:1588‐1598. 31862959 10.1038/s41375-019-0682-7 PMC7272263

[31] Leal AS , Liu P , Krieger‐Burke T , Ruggeri B , Liby KT . The bromodomain inhibitor, INCB057643, targets both cancer cells and the tumor microenvironment in two preclinical models of pancreatic cancer. Cancers (Basel). 2020;13:96. 33396954 10.3390/cancers13010096 PMC7794921

[32] Dura B , Choi J‐Y , Zhang K , et al. scFTD‐seq: freeze‒thaw lysis based, portable approach toward highly distributed single‐cell 3′ mRNA profiling. Nucleic Acids Res. 2019;47:e16. 30462277 10.1093/nar/gky1173 PMC6379653

[33] Matsumoto M , Tanaka T , Kaisho T , et al. A novel LPS‐inducible C‐type lectin is a transcriptional target of NF‐IL6 in macrophages. J Immunol. 1999;163:5039‐5048. 10528209

[34] Schoenen H , Huber A , Sonda N , et al. Differential control of Mincle‐dependent cord factor recognition and macrophage responses by the transcription factors C/EBPβ and HIF1α. J Immunol. 2014;193:3664‐3675. 25156364 10.4049/jimmunol.1301593

[35] Seifert L , Werba G , Tiwari S , et al. The necrosome promotes pancreatic oncogenesis via CXCL1 and Mincle‐induced immune suppression. Nature. 2016;532:245‐249. 27049944 10.1038/nature17403 PMC4833566

[36] Zhang Y , Wei H , Fan L , et al. CLEC4s as potential therapeutic targets in hepatocellular carcinoma microenvironment. Front Cell Dev Biol. 2021;9:681372. 34409028 10.3389/fcell.2021.681372 PMC8367378

[37] Jiang Q , Xiao D , Wang A , et al. CLEC4E upregulation in gastric cancer: a potential therapeutic target correlating with tumor‐associated macrophages. Heliyon. 2024;10:e27172. 38463883 10.1016/j.heliyon.2024.e27172 PMC10920739

[38] Yin S , Dai W , Kuang T , et al. Punicalagin promotes Mincle‐mediated phagocytosis of macrophages via the NF‐κB and MAPK signaling pathways. Eur J Pharmacol. 2024;970:176435. 38428663 10.1016/j.ejphar.2024.176435

[39] Clément M , Basatemur G , Masters L , et al. Necrotic cell sensor Clec4e promotes a proatherogenic macrophage phenotype through activation of the unfolded protein response. Circulation. 2016;134:1039‐1051. 27587433 10.1161/CIRCULATIONAHA.116.022668

[40] Guerrero‐Juarez CF , Schilf P , Li J , et al. C‐type lectin receptor expression is a hallmark of neutrophils infiltrating the skin in epidermolysis bullosa acquisita. Front Immunol. 2023;14:1266359. 37799716 10.3389/fimmu.2023.1266359 PMC10548123

[41] Hasgur S , Yamamoto Y , Fan R , et al. Macrophage‐inducible C‐type lectin activates B cells to promote T cell reconstitution in heart allograft recipients. Am J Transplant. 2022;22:1779‐1790. 35294793 10.1111/ajt.17033 PMC9296143

[42] Mohanraj M , Sekar P , Liou H‐H , Chang S‐F , Lin W‐W . The mycobacterial adjuvant analogue TDB attenuates neuroinflammation via Mincle‐independent PLC‐γ1/PKC/ERK signaling and microglial polarization. Mol Neurobiol. 2019;56:1167‐1187. 29876879 10.1007/s12035-018-1135-4

[43] Martínez‐López M , Iborra S , Conde‐Garrosa R . Microbiota sensing by Mincle‐Syk axis in dendritic cells regulates interleukin‐17 and ‐22 production and promotes intestinal barrier integrity. Immunity. 2019;50:446‐461.e9. 30709742 10.1016/j.immuni.2018.12.020 PMC6382412

[44] Boi M , Gaudio E , Bonetti P , et al. The BET bromodomain inhibitor OTX015 affects pathogenetic pathways in preclinical B‐cell tumor models and synergizes with targeted drugs. Clin Cancer Res. 2015;21:1628‐1638. 25623213 10.1158/1078-0432.CCR-14-1561

[45] Delmore JE , Issa GC , Lemieux ME , et al. BET bromodomain inhibition as a therapeutic strategy to target c‐Myc. Cell. 2011;146:904‐917. 21889194 10.1016/j.cell.2011.08.017 PMC3187920

[46] Ott CJ , Kopp N , Bird L , et al. BET bromodomain inhibition targets both c‐Myc and IL7R in high‐risk acute lymphoblastic leukemia. Blood. 2012;120:2843‐2852. 22904298 10.1182/blood-2012-02-413021 PMC3466965

[47] Stathis A , Bertoni F . BET proteins as targets for anticancer treatment. Cancer Discov. 2018;8:24‐36. 29263030 10.1158/2159-8290.CD-17-0605

[48] Adamo H , Hammarsten P , Hägglöf C , et al. Prostate cancer induces C/EBPβ expression in surrounding epithelial cells which relates to tumor aggressiveness and patient outcome. Prostate. 2019;79:435‐445. 30536410 10.1002/pros.23749

[49] Matsuda T , Kido Y , Asahara S , et al. Ablation of C/EBPbeta alleviates ER stress and pancreatic beta cell failure through the GRP78 chaperone in mice. J Clin Invest. 2010;120:115‐126. 19955657 10.1172/JCI39721 PMC2798684

[50] Qi H , Zheng Z , Liu Q . Activation of BZW1 by CEBPB in macrophages promotes eIF2α phosphorylation‐mediated metabolic reprogramming and endoplasmic reticulum stress in MRL/lpr lupus‐prone mice. Cell Mol Biol Lett. 2023;28:79. 37828427 10.1186/s11658-023-00494-1 PMC10571419

[51] Staiger J , Lueben MJ , Berrigan D , et al. C/EBPbeta regulates body composition, energy balance‐related hormones and tumor growth. Carcinogenesis. 2009;30:832‐840. 19056928 10.1093/carcin/bgn273 PMC2675647

[52] van der Krieken SE , Popeijus HE , Mensink RP , Plat J . CCAAT/enhancer binding protein β in relation to ER stress, inflammation, and metabolic disturbances. Biomed Res Int. 2015;2015:324815. 25699273 10.1155/2015/324815 PMC4324884

[53] Yan C , Zhu M , Staiger J , Johnson PF , Gao H . C5a‐regulated CCAAT/enhancer‐binding proteins β and δ are essential in Fcγ receptor‐mediated inflammatory cytokine and chemokine production in macrophages. J Biol Chem. 2012;287:3217‐3230. 22147692 10.1074/jbc.M111.280834 PMC3270976

[54] Yang Y , Jin X , Xie Y , et al. The CEBPB+ glioblastoma subcluster specifically drives the formation of M2 tumor‐associated macrophages to promote malignancy growth. Theranostics. 2024;14:4107‐4126. 38994023 10.7150/thno.93473 PMC11234274

[55] Hu Z , Sui Q , Jin X , et al. IL6‐STAT3‐C/EBPβ‐IL6 positive feedback loop in tumor‐associated macrophages promotes the EMT and metastasis of lung adenocarcinoma. J Exp Clin Cancer Res. 2024;43:63. 38424624 10.1186/s13046-024-02989-x PMC10903044

