# Supplementary material for: Targeting CLEC4E in immunosuppressive tumour‐associated macrophages via BET inhibition
Source: Clin Transl Med. 2025 Oct 15;15(10):e70505. doi: 10.1002/ctm2.70505 (PMC12521789; doi:10.1002/ctm2.70505)
Supplement: Supplementary file 1 — Supporting Information [file CTM2-15-e70505-s001.docx]

**Figure S1.** Supplementary to Figure 1. (a) Expression of M2 markers (Arg1, CD163, CD206) and M1 markers (CD86, iNOS, CD40) assessed in TAMs induced from peritoneal macrophages. (b) Expression of CLEC4E in skin carcinoma and normal skin tissues from TCGA dataset. (c) Association between CLEC4E and macrophage or tumor cell markers. (d) Overall survival analysis of patients with high and low CD68^+^ infiltration. Median CD68^+^ infiltration level was determined as the cutoff. (e) Overall survival analysis of patients with high and low CLEC4E^+^ infiltration within high CD68^+^ patients. Median CLEC4E^+^ infiltration level was determined as the cutoff.

**Figure S2.** Supplementary to Figure 2. (a) Knockout strategy of CLEC4E in conditional knockout mouse. LoxP was inserted between exon 2 and 3, and exon 5 and 6. Exon 3-5 was deleted after cre recombination. (b) Generation of CLEC4E flox/flox Lyz2 cre mouse. (c) Identification of CLEC4E flox, lyz2cre mutation and wildtype allele. (d) RT-PCR of CLEC4E with macrophage, T cells and muscle cells from mice with different genotypes. (e) Body weight of CLEC4E conditional knockout and control mice in both genders. (f) Flow cytometry analysis of the CD206 high and low cells within CD68^+^ Macrophages from melanoma tissues at day 10. (g) Flow cytometry analysis of the CD206 high and low cells within CD68^+^ Macrophages from ovarian model ascites at week 8

**Figure S3.** Supplementary to Figure 3. (a) UMAP plot of total cells from B16F10 melanoma tissues. (b) Dot plot of gene markers for cluster identification. (c) UMAP of representative gene markers of each cluster. (d) Bar chart of proportions of tumor and non-tumor cells in two groups of mice. (e) Dot plot of gene markers for macrophage subclusters. (f) UMAP of myeloid cell population and representative markers for macrophages and dendritic cells. (g) Expression of Clec4e in UMAP of myeloid cell population. (h) UMAP of the distribution of cells in 5 states and gene markers of Clec4 family. State 1 and 2, state 4 and 5 were aggregated. (i) Expression of Clec4e in myeloid cell population. (j) GO enrichment analysis of signaling pathways up-regulated in state 1 versus state 2, and in state 4 versus state 5. (k) qRT-PCR of peritoneal macrophages from CLEC4E knockout and control mice.

Supplementary Table 1. Sequence of primers used in RT-PCR

| Name (mouse primer) | Sequence (5’ - 3’) |
| --- | --- |
| Gapdh-Forward | CATCACTGCCACCCAGAAGACTG |
| Gapdh-Reverse | ATGCCAGTGAGCTTCCCGTTCAG |
| Actb-Forward | CATTGCTGACAGGATGCAGAAGG |
| Actb-Reverse | TGCTGGAAGGTGGACAGTGAGG |
| Clec4e-Forward | AGTGCTCTCCTGGACGATAG |
| Clec4e-Reverse | CCTGATGCCTCACTGTAGCAG |
| Ccl8-Forward | GGGTGCTGAAAAGCTACGAGAG |
| Ccl8-Reverse | GGATCTCCATGTACTCACTGACC |
| Cd74-Forward | GCTGGATGAAGCAGTGGCTCTT |
| Cd74-Reverse | GATGTGGCTGACTTCTTCCTGG |
| H2k1-Forward | GGCAATGAGCAGAGTTTCCGAG |
| H2k1-Reverse | CCACTTCACAGCCAGAGATCAC |
| Il1b-Forward | TGGACCTTCCAGGATGAGGACA |
| Il1b-Reverse | GTTCATCTCGGAGCCTGTAGTG |
| Cxcl9-Forward | CCTAGTGATAAGGAATGCACGATG |
| Cxcl9-Reverse | CTAGGCAGGTTTGATCTCCGTTC |
| Fcgr4-Forward | TGACAGTGGCTCCTACTTCTGC |
| Fcgr4-Reverse | GAGTCCTATCAGCAGGCAGAATG |
| Fcgrt-Forward | CATTGCTGGAGGTCAAACGTGG |
| Fcgrt-Reverse | CGATTCCAACCACAGGCACAGA |
| H2ab1-Forward | GTGTGCAGACACAACTACGAGG |
| H2ab1-Reverse | CTGTCACTGAGCAGACCAGAGT |
| H2aa-Forward | GGAGGTGAAGACGACATTGAGG |
| H2aa-Reverse | CTCAGGAAGCATCCAGACAGTC |
| Mki67-Forward | GAGGAGAAACGCCAACCAAGAG |
| Mki67-Reverse | TTTGTCCTCGGTGGCGTTATCC |
| Ccna2-Forward | TTGTAGGCACGGCTGCTATGCT |
| Ccna2-Reverse | GGTGCTCCATTCTCAGAACCTG |
| Ccnd1-Forward | GCAGAAGGAGATTGTGCCATCC |
| Ccnd1-Reverse | AGGAAGCGGTCCAGGTAGTTCA |
| Mcm2-Forward | CCGTTCCAAGGATGCCATTCTC |
| Mcm2-Reverse | TGGAAAGCCGTTGGCGGTGTTA |
| Cdk1-Forward | CATGGACCTCAAGAAGTACCTGG |
| Cdk1-Reverse | CAAGTCTCTGTGAAGAACTCGCC |
| Cd86-Forward | ACGTATTGGAAGGAGATTACAGCT |
| Cd86-Reverse | TCTGTCAGCGTTACTATCCCGC |
| Brd2-Forward | AATGGCTTCTGTACCAGCTTTAC |
| Brd2-Reverse | CTGGCTTTTTGGGATTGGACA |
| Brd3-Forward | GGGCGAAAGACTAACCAACTG |
| Brd3-Reverse | GAAAGGCCAGGCAAACTGATG |
| Brd4-Forward | CCTCCCAAATGTCTACAACGC |
| Brd4-Reverse | GAGCAGATATTGCAGTTGGTT |

Supplementary Table 2. Antibodies used in western blot, immunostaining and flow cytometry

| **Protein name** | **Clone** | **Company** | **CatLog No** | **Species** | **Application** |
| --- | --- | --- | --- | --- | --- |
| CD68 | EPR20545 | Abcam | Ab213363 | Human | IF |
| CD68 | FA-11 | Abcam | Ab53444 | Mouse | IF |
| Mincle(CLEC4E) | 4A9 | MBLbio | D292-3 | Mouse | WB |
| Mincle(CLEC4E) | B-7 | Santa Cruz | sc-390806 | Human  Mouse | WB, IF  IF |
| Ki67 | SolA15 | eBioscience | 11-5698-82 | Mouse | IF |
| Erk1/2 | 137F5 | Cell signalling | 4695 | Mouse | WB |
| p-Erk1/2 (Thr202/Tyr204) | D13.14.4E | Cell signalling | 4370 | Mouse | WB |
| PLCγ2 | N.A. | Cell signalling | 3872 | Mouse | WB |
| p-PLCγ2  (Tyr1217) | N.A. | Cell signalling | 3871 | Mouse | WB |
| Syk | N.A. | Cell signalling | 2712 | Mouse | WB |
| p-Syk | C87C1 | Cell signalling | 2710 | Mouse | WB |
| Granzyme B | EPR22645-206 | Abcam | ab255598 | Mouse | IHC |
| CEBPB | N.A. | Proteintech | 23431-1-AP | Mouse, human | WB |
| Vinculin | 2B5A7 | Proteintech | 66305-1-Ig | Mouse | WB |
| GAPDH | 1E6D9 | Proteintech | 60004-1-Ig | Mouse | WB |
| α-Tubulin | N.A. | Proteintech | 11224-1-AP | Mouse, human | WB |
| β-actin | C4 | Santa Cruz | sc-47778 | Human | WB |
| CD45 | 30-F11 | Biolegend | 103115 | Mouse | FC |
| CD11b | M1/70 | Biolegend | 101207 | Mouse | FC |
| F4/80 | BM8 | Biolegend | 123115 | Mouse | FC |
| CD68 | FA-11 | Biolegend | 137009 | Mouse | FC |
| CD206 | C068C2 | Biolegend | 141705 | Mouse | FC |
| CD3 | 17A2 | Biolegend | 100236 | Mouse | FC |
| CD8 | 53-6.7 | Biolegend | 100722 | Mouse | FC |
| CD4 | GK1.5 | Biolegend | 100434 | Mouse | FC |
| Granzyme B | QA16A02 | Biolegend | 372203 | Mouse | FC |
| Ki67 | 16A8 | Biolegend | 652403 | Mouse | FC |
| Viability dye | N.A. | eBioscience | 65-0865-14 | Mouse | FC |
