# Supplementary material for: Targeting CLEC4E in immunosuppressive tumour‐associated macrophages via BET inhibition
Source: Clin Transl Med. 2025 Oct 15;15(10):e70505. doi: 10.1002/ctm2.70505 (PMC12521789; doi:10.1002/ctm2.70505)
Supplement: Supplementary file 2 — Supporting Information [file CTM2-15-e70505-s002.docx]

Supplementary Table 1. Sequence of primers used in RT-PCR

| Name (mouse primer) | Sequence (5’ - 3’) |
| --- | --- |
| Gapdh-Forward | CATCACTGCCACCCAGAAGACTG |
| Gapdh-Reverse | ATGCCAGTGAGCTTCCCGTTCAG |
| Actb-Forward | CATTGCTGACAGGATGCAGAAGG |
| Actb-Reverse | TGCTGGAAGGTGGACAGTGAGG |
| Clec4e-Forward | AGTGCTCTCCTGGACGATAG |
| Clec4e-Reverse | CCTGATGCCTCACTGTAGCAG |
| Ccl8-Forward | GGGTGCTGAAAAGCTACGAGAG |
| Ccl8-Reverse | GGATCTCCATGTACTCACTGACC |
| Cd74-Forward | GCTGGATGAAGCAGTGGCTCTT |
| Cd74-Reverse | GATGTGGCTGACTTCTTCCTGG |
| H2k1-Forward | GGCAATGAGCAGAGTTTCCGAG |
| H2k1-Reverse | CCACTTCACAGCCAGAGATCAC |
| Il1b-Forward | TGGACCTTCCAGGATGAGGACA |
| Il1b-Reverse | GTTCATCTCGGAGCCTGTAGTG |
| Cxcl9-Forward | CCTAGTGATAAGGAATGCACGATG |
| Cxcl9-Reverse | CTAGGCAGGTTTGATCTCCGTTC |
| Fcgr4-Forward | TGACAGTGGCTCCTACTTCTGC |
| Fcgr4-Reverse | GAGTCCTATCAGCAGGCAGAATG |
| Fcgrt-Forward | CATTGCTGGAGGTCAAACGTGG |
| Fcgrt-Reverse | CGATTCCAACCACAGGCACAGA |
| H2ab1-Forward | GTGTGCAGACACAACTACGAGG |
| H2ab1-Reverse | CTGTCACTGAGCAGACCAGAGT |
| H2aa-Forward | GGAGGTGAAGACGACATTGAGG |
| H2aa-Reverse | CTCAGGAAGCATCCAGACAGTC |
| Mki67-Forward | GAGGAGAAACGCCAACCAAGAG |
| Mki67-Reverse | TTTGTCCTCGGTGGCGTTATCC |
| Ccna2-Forward | TTGTAGGCACGGCTGCTATGCT |
| Ccna2-Reverse | GGTGCTCCATTCTCAGAACCTG |
| Ccnd1-Forward | GCAGAAGGAGATTGTGCCATCC |
| Ccnd1-Reverse | AGGAAGCGGTCCAGGTAGTTCA |
| Mcm2-Forward | CCGTTCCAAGGATGCCATTCTC |
| Mcm2-Reverse | TGGAAAGCCGTTGGCGGTGTTA |
| Cdk1-Forward | CATGGACCTCAAGAAGTACCTGG |
| Cdk1-Reverse | CAAGTCTCTGTGAAGAACTCGCC |
| Cd86-Forward | ACGTATTGGAAGGAGATTACAGCT |
| Cd86-Reverse | TCTGTCAGCGTTACTATCCCGC |
| Brd2-Forward | AATGGCTTCTGTACCAGCTTTAC |
| Brd2-Reverse | CTGGCTTTTTGGGATTGGACA |
| Brd3-Forward | GGGCGAAAGACTAACCAACTG |
| Brd3-Reverse | GAAAGGCCAGGCAAACTGATG |
| Brd4-Forward | CCTCCCAAATGTCTACAACGC |
| Brd4-Reverse | GAGCAGATATTGCAGTTGGTT |
