# Supplementary material for: Targeting CLEC4E in immunosuppressive tumour‐associated macrophages via BET inhibition
Source: Clin Transl Med. 2025 Oct 15;15(10):e70505. doi: 10.1002/ctm2.70505 (PMC12521789; doi:10.1002/ctm2.70505)
Supplement: Supplementary file 3 — Supporting Information [file CTM2-15-e70505-s003.docx]

Supplementary Table 1. Antibodies used in western blot, immunostaining and flow cytometry

| **Protein name** | **Clone** | **Company** | **CatLog No** | **Species** | **Application** |
| --- | --- | --- | --- | --- | --- |
| CD68 | EPR20545 | Abcam | Ab213363 | Human | IF |
| CD68 | FA-11 | Abcam | Ab53444 | Mouse | IF |
| Mincle | 4A9 | MBLbio | D292-3 | Mouse | WB |
| Mincle | B-7 | Santa Cruz | sc-390806 | Human  Mouse | WB, IF  IF |
| Ki67 | SolA15 | eBioscience | 11-5698-82 | Mouse | IF |
| Erk1/2 | 137F5 | Cell signalling | 4695 | Mouse | WB |
| p-Erk1/2 (Thr202/Tyr204) | D13.14.4E | Cell signalling | 4370 | Mouse | WB |
| Granzyme B | EPR22645-206 | Abcam | ab255598 | Mouse | IHC |
| CEBPB | N.A. | Proteintech | 23431-1-AP | Mouse, human | WB |
| Vinculin | 2B5A7 | Proteintech | 66305-1-Ig | Mouse | WB |
| GAPDH | 1E6D9 | Proteintech | 60004-1-Ig | Mouse | WB |
| α-Tubulin | N.A. | Proteintech | 11224-1-AP | Mouse, human | WB |
| β-actin | C4 | Santa Cruz | sc-47778 | Human | WB |
| CD45 | 30-F11 | Biolegend | 103115 | Mouse | FC |
| CD11b | M1/70 | Biolegend | 101207 | Mouse | FC |
| F4/80 | BM8 | Biolegend | 123115 | Mouse | FC |
| CD68 | FA-11 | Biolegend | 137009 | Mouse | FC |
| CD206 | C068C2 | Biolegend | 141705 | Mouse | FC |
| CD3 | 17A2 | Biolegend | 100236 | Mouse | FC |
| CD8 | 53-6.7 | Biolegend | 100722 | Mouse | FC |
| CD4 | GK1.5 | Biolegend | 100434 | Mouse | FC |
| Granzyme B | QA16A02 | Biolegend | 372203 | Mouse | FC |
| Ki67 | 16A8 | Biolegend | 652403 | Mouse | FC |
| Viability dye | N.A. | eBioscience | 65-0865-14 | Mouse | FC |
